# Silicon doping-engineered asymmetric coordination and electronic structure modulation for amplified single-atom nanozyme catalytic therapy

**DOI:** 10.1016/j.mtbio.2026.103082

**Published:** 2026-04-03

**Authors:** Bo Liu, Huihan Yi, Zhan Zhuang, Penghui Wei, Hongjia Zheng, Huimin Wang, Yifei Tu, Jinbao Xie, Yang Zhu, Xu Li

**Affiliations:** a Fujian Key Laboratory of Precision Medicine for Cancer, Department of Thoracic Surgery, The First Affiliated Hospital, Fujian Medical University, Fuzhou, 350005, China; b Department of Thoracic Surgery National Regional Medical Center, Binhai Campus of the First Affiliated Hospital, Fujian Medical University, Fuzhou, 350212, China; c Department of Neurosurgery, Neurosurgery Research Institute, The First Affiliated Hospital, Fujian Medical University, Fuzhou, 350005, China; d Department of Neurosurgery, National Regional Medical Center, Binhai Campus of the First Affiliated Hospital, Fujian Medical University, Fuzhou, 350212, China

**Keywords:** Single-atom nanozyme, Ferroptosis, Catalytic therapy, Asymmetric coordination, Heteroatom-doping

## Abstract

Single-atom nanozymes (SANs), which feature tunable electronic structures and optimized atomic utilization efficiency, have garnered significant attention in biomedical applications. Despite substantial advancements, the catalytic performance of SANs remains suboptimal compared with that of natural enzymes, largely due to their symmetric coordination environments and electronic structures. Herein, we successfully engineer an asymmetrically coordinated Fe-based SAN with Si doping, termed Fe-SiN_3_/SAN, which demonstrates superior catalytic performance compared with its symmetric counterpart, Fe-N_4_/SAN. The low electronegativity of Si induces slight elongation of the Fe-N bonds in Fe-SiN_3_/SAN, optimizing the adsorption and desorption of oxygen intermediates and thereby significantly enhancing catalytic activity. Density functional theory (DFT) calculations reveal that asymmetric coordination in Fe-SiN_3_/SAN enhances structural electron activation, shifting the d-band center of Fe closer to the Fermi level. This shift facilitates the adsorption and activation of hydrogen peroxide and glutathione. Importantly, Bader charge analysis based on DFT calculations reveals that Fe-SiN_3_/SAN exhibits a lower charge during the desorption of rate-determining intermediates (∗OH and ∗GS) compared with Fe-N_4_/SAN, confirming its superior reactive oxygen species generation capability. Experimental results further confirm that Fe-SiN_3_/SAN effectively induces irreversible tumor ferroptosis by promoting lipid peroxidation accumulation and inactivating glutathione peroxidase 4.

## Graphical abstract

We engineered a Si-doped asymmetric Fe-based single-atom nanozyme (Fe-SiN_3_/SAN) that exhibited superior enzymatic activity compared to symmetric Fe-N_4_/SAN. The low electronegativity of Si elongates the Fe-N bonds, optimizing oxygen intermediate adsorption/desorption. Density functional theory calculations revealed that asymmetric coordination facilitated the activation of hydrogen peroxide and glutathione, thus confirming its enhanced catalytic activity.

**Figure:**
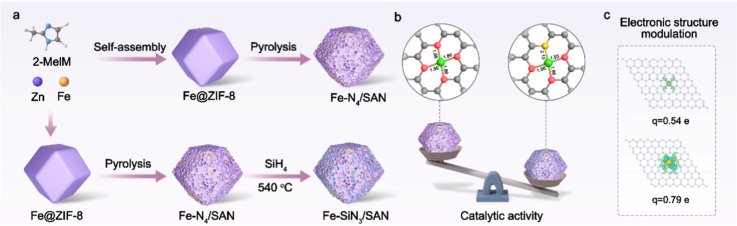

## 1 Introduction

The advent of next-generation single-atom nanozymes (SANs) represents a transformative leap forward, offering enhanced biomedical applicability through their tunable electronic properties and optimized atomic utilization efficiency [[1], [2], [3], [4], [5]]. Among the various SANs developed in recent years, N-doped C (N-C) immersed Fe-based nanozymes, which feature symmetric Fe–N_4_ active sites, have emerged as a focal point of intensive research [[6], [7], [8], [9], [10]]. Despite their promising potential, these Fe–N_4_ SANs still lag behind benchmark natural enzymes in terms of catalytic performance [4,11,12]. This shortfall is primarily attributed to their symmetric electron distribution, which limits the adsorption strength of critical oxygen intermediates (e.g., O∗, OH∗, and OOH∗) [[13], [14], [15]]. Disrupting this inherently symmetric charge distribution has been identified as a key strategy to optimize the adsorption strength of critical oxygen intermediates [[16], [17], [18]]. Particularly noteworthy is the symmetrical Fe–N_4_/SAN with single e_g_ electron filling, which has been widely acclaimed by the scientific community as an ideal nanozyme [19,20]. This recognition is largely because of its impressive capacity for reactive oxygen species (ROS) generation [21,22]. To overcome the current limitations and unlock the full potential of these nanozymes, the local coordination environments surrounding Fe–N_4_ SANs must be modulated [23]. Such optimization enables optimization of the asymmetric charge distribution, thereby enhancing both the reactivity and selectivity of SANs.

Fe-based SANs featuring sufficiently isolated Fe–N_4_ active sites have demonstrated superior peroxidase (POD)- and glutathione oxidase (GSHOX)-mimicking activities compared with ferric oxide (Fe_2_O_3_) nanoparticles [[24], [25], [26]]. However, these Fe–N_4_ coordination structures are hampered by their weak adsorption of hydrogen peroxide (H_2_O_2_) and GSH, leading to the premature detachment of intermediate species [11]. To overcome this challenge, an effective strategy is to regulate the coordination environments of the isolated Fe active sites [27,28]. This can enhance the adsorption and dissociation of H_2_O_2_ at the active sites, thereby enhancing the POD-like activity of Fe-based SANs. A promising approach to boost these absorption and dissociation capabilities is to introduce low-electronegativity heteroatoms [[29], [30], [31], [32]], such as S [[33], [34], [35]], P [20,36], or B [19,37,38], into the coordination environment of the central metal atoms. Previous research has shown that heteroatom doping in carbon substrates can rationally regulate the electronic structure and local environment of metal centers in SANs [39]. This modification not only amplifies catalytic activity but also optimizes the adsorption/desorption behavior of oxygen intermediates, thereby further strengthening the catalytic performance [40,41]. Notably, Si has been identified as having the lowest electronegativity among the heteroatoms S, P, and B. Si doping can change the Fe–N bond length, which helps to immobilize Fe atoms, thereby enhancing both catalytic stability and efficiency [42,43]. To the best of our knowledge, leveraging Si-doping as a predictive descriptor to guide the design of Fe-based SANs for catalytic therapy represents a nascent and largely uncharted frontier in contemporary research.

**Scheme 1:**
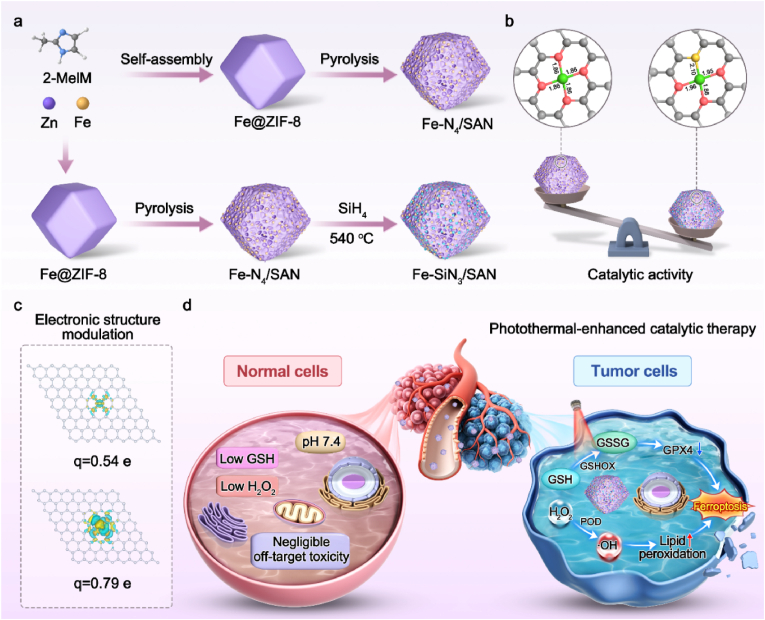
**Illustration of the catalytic therapy mechanism enhanced by silicon doping.** (a) Synthetic schematic of Fe–N_4_/SAN and Fe–SiN_3_/SAN. (b) The catalytic activity and bond length of Fe–N_4_/SAN and Fe–SiN_3_/SAN. (c) Bader charge analysis of Fe–N_4_/SAN and Fe–SiN_3_/SAN for electronic structure modulation. (d) Schematic of catalytic therapy-induced tumor ferroptosis.

In this study, we successfully engineered two distinct coordination environments: symmetric coordination with isolated Fe–N_4_ active sites and asymmetric coordination with isolated Fe–SiN_3_ active sites (Scheme 1). The incorporation of Si into Fe–SiN_3_/SAN, characterized by its large geometric radius, induces significant geometric distortion and electronic structure modulation through the stretching of the Fe–N bond. This distortion effectively balances the adsorption and desorption of oxygen intermediates, thereby significantly strengthening the catalytic activity. Both Fe–N_4_/SAN and Fe–SiN_3_/SAN exhibit remarkable POD- and GSHOx-mimicking activities, effectively converting H_2_O_2_ into hydroxyl radicals (•OH) and oxidizing GSH into glutathione disulfide (GSSG). Notably, Fe–SiN_3_/SAN demonstrates superior POD- and GSHOx-mimicking activities compared to its Fe–N_4_/SAN counterparts, highlighting that Si coordination significantly enhances the transfer of electrons and optimizes the adsorption energy of the reaction intermediates. Density functional theory (DFT) calculations revealed that the asymmetric Fe–SiN_3_/SAN configuration enhances the activation ability of structural electrons around the Fe center, increases the number of unpaired electrons, and brings the d-band center of the Fe atoms closer to the Fermi level. This configuration facilitates H_2_O_2_ and GSH adsorption, thereby accelerating the rate-determining step of the catalytic reaction rate-determining step (RDS). Furthermore, Si coordination slightly stretches the Fe–N bond length, independently optimizing charge transfer between the Fe active sites and intermediates, which lowers the energy barrier. Importantly, DFT-derived Bader charge analysis revealed that asymmetric Fe–SiN_3_/SAN had a lower charge of desorption for the RDS intermediates ∗OH and ∗GS compared to symmetric Fe–N_4_/SAN, confirming its superior ROS generation capability. The experimental results demonstrated that Fe–SiN_3_/SAN effectively induces irreversible tumor ferroptosis via lipid peroxidation (LPO) accumulation and glutathione peroxidase 4 (GPX4) inactivation. Additionally, under 808 nm laser irradiation, the catalytic activities of the nanozymes were further boosted, leading to significant inhibition of tumor growth. This study provides an efficient method for tuning the adsorption/desorption behavior of intermediates on SANs to improve their catalytic performance, thereby offering a promising approach for biomedical applications.

## 2 Results and discussion

**Fig. 1:**
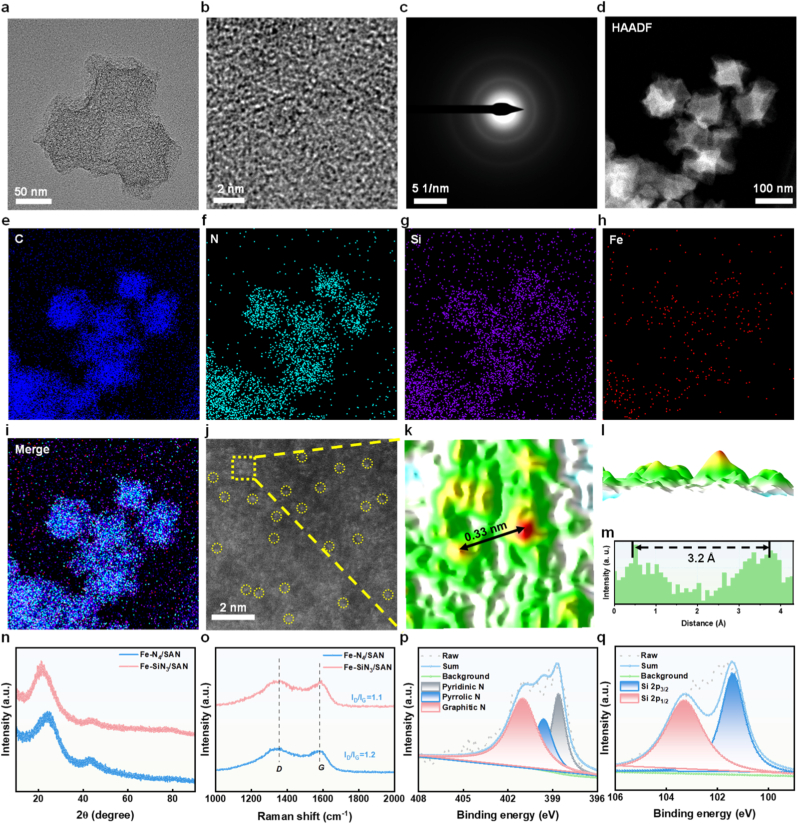
(a) TEM, (b) HR TEM, (c) SAED images of Fe–SiN_3_/SAN. (d–i) HAADF-STEM images of Fe–SiN_3_/SAN. (j) AC HAADF-STEM images of Fe–SiN_3_/SAN. The isolated Fe atoms are encircled in yellow. (k–l) 3D model of Fe–SiN_3_/SAN bright spots along the yellow dotted line, and (m) the corresponding intensity profiles along the black line in ∗1. (n) XRD and (o) Raman patterns of Fe–SiN_3_/SAN and Fe–N_4_/SAN. (p) High-resolution XPS of N 1s and (q) Si 2p.

The synthesis route for the Fe-based SANs is illustrated in Scheme 1. First, the Fe@ZIF-8 precursor, which retained a rhombic dodecahedron morphology (Fig. S1), was annealed to produce Fe–N_4_/SAN at 950 °C for 3 h in an argon atmosphere (Fig. S2). In contrast, Fe–SiN_3_/SAN was fabricated using SiH_4_ gas as the Si resource. Transmission electron microscopy (TEM) images revealed that Fe–SiN_3_/SAN had a uniform dodecahedral shape with an approximate diameter of 100 nm (Fig. 1a). High-resolution TEM further revealed that the surfaces of Fe–SiN_3_/SAN and Fe–N_4_/SAN became rough and porous after the high-temperature carbonization (Fig. 1b and S3). The selected area electron diffraction (SAED) image of Fe–SiN_3_/SAN displays two diffuse diffraction rings, indicative of amorphous N–C shells (Fig. 1c). Energy-dispersive X-ray spectroscopy (EDX) mapping demonstrates a uniform distribution of Fe, C, N, and Si atoms within Fe–SiN_3_/SAN (Fig. 1d–i). Aberration-corrected atomic-resolution high-angle annular dark-field scanning transmission electron microscopy (HAADF-STEM) directly revealed abundant bright dots, encircled in yellow, corresponding to atomically dispersed Fe (Fig. 1j). The inter-atomic distance histogram indicates that the distance between the two single-atomic Fe is 3.2 Å, which matches the expected spacing for isolated Fe atoms, thus confirming the exclusive presence of atomically dispersed Fe (Fig. 1k–m). X-ray diffraction (XRD) patterns showed no crystalline peaks corresponding to metallic Fe or Fe oxide nanoparticles, suggesting minimal Fe aggregation in Fe–SiN_3_/SAN and thus supporting the successful formation of Fe-based SANs (Fig. 1m). Furthermore, Raman analysis demonstrated that the N-C shell was poorly crystallized post-pyrolysis, with a high density of defects, which aided in stabilizing the atomically dispersed Fe atoms (Fig. 1n). Collectively, these findings confirmed the successful synthesis of Fe-based SANs.

**Fig. 2:**
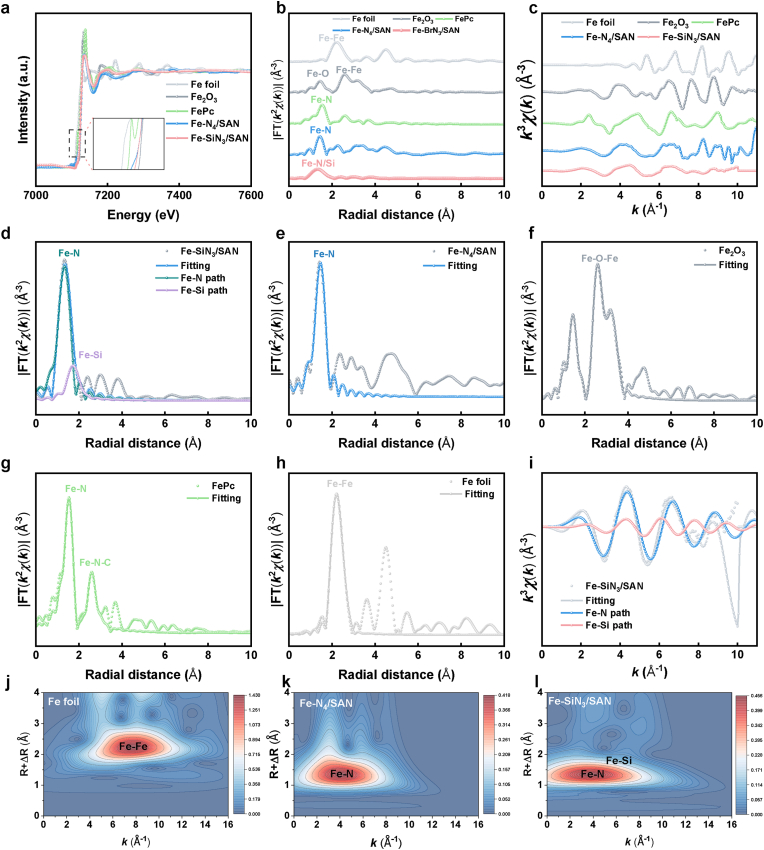
(a) XANES spectra and matching (b) Fourier transform EXAFS spectra of Fe–SiN_3_/SAN and Fe–N_4_/SAN. (c) EXAFS curves of Fe–SiN_3_/SAN and Fe–N_4_/SAN at the k space. (d-h) EXAFS fitting curve of Fe–SiN_3_/SAN, Fe–N_4_/SAN, Fe_2_O_3_, FePc, and Fe foil at the R space. (i) EXAFS fitting curve of Fe–SiN_3_/SAN at the k space. (j-l) WT of Fe foil, Fe–SiN_3_/SAN, and Fe–N_4_/SAN.

X-ray photoelectron spectroscopy (XPS) analysis was performed to elucidate the binding states of the Fe, Si, C, and N atoms in Fe–SiN_3_/SAN. The Fe 2p spectrum of Fe–SiN_3_/SAN exhibited a slight shift toward a lower binding energy, confirming that partial electrons were transferred from the Si atoms to the central Fe atoms via the N atoms (Fig. S4). As depicted in Fig. S5, the C 1s spectrum of Fe–SiN_3_/SAN predominantly features peaks corresponding to sp^2^-hybridized graphitic C, which can be attributed to C–C and C–N/Si bonding configurations. The N 1s spectrum shows three distinct N species: graphitic N, pyrrolic N, and pyridinic N. These nitrogen functionalities provide coordination sites for anchoring isolated Fe atoms, potentially enhancing the catalytic performance (Fig. 1p). The high-resolution Si 2p spectrum in Fe–SiN_3_/SAN yields two peaks centered at approximately 101.4 and 103.3 eV (Fig. 1q), corresponding to Fe–Si and N–Si bonds, respectively. To further elucidate the Fe coordination environment, synchrotron-radiation-based X-ray absorption near-edge structure (XANES) and extended X-ray absorption fine structure (EXAFS) analyses were conducted. The Fe K-edge XANES spectra of both Fe–SiN_3_/SAN and Fe–N_4_/SAN display absorption edge energy intermediate between those of Fe foil and Fe_2_O_3_ (Fig. 2a), indicating an Fe^δ+^ (0 <δ < 3) oxidation state, which is consistent with the XPS findings. The Fourier transform magnitude of the Fe–N_4_/SAN EXAFS (R-space) exhibits a distinct peak at 1.44 Å, assignable to the Fe–N bonds. In contrast, Fe–SiN_3_/SAN EXAFS shows a peak at 1.35 Å, assignable to overlapping contributions from both Fe–N and Fe–Si bonds. Notably, no significant peak was observed around 2.18 Å (a characteristic of Fe–Fe bonds), further confirming the atomic dispersion of Fe in the Fe-based SANs (Fig. 2b and c). EXAFS fitting analysis at the Fe K-edge provided definitive structural parameters: Fe atoms in Fe–N_4_/SAN were coordinated with four N atoms, whereas those in Fe–SiN_3_/SAN adopted a 1:3 coordination with Si and N atoms (Fig. 2d–i, S6, and Table S1). Wavelet transform (WT) analysis was used to corroborate the atomic dispersion of the Fe species. As anticipated, Fe–SiN_3_/SAN exhibits WT signals at 3.89 Å^−1^ and 6.19 Å^−1^, assignable to the Fe–N and Fe–Si bonds, respectively, with no discernible signal for Fe–Fe bonds (Fig. 2j–l and S7). Furthermore, our particle size monitoring results robustly confirm that Fe-SiN_3_/SAN exhibited excellent stability in a blood matrix (Fig. S8). we performed accelerated stability tests under simulated acidic tumor conditions (High level of GSH). The results reveal that despite the slightly elongated Fe-N bond, Si doping enhances the overall structural robustness of the single-atom Fe center in acidic environments (Fig. S9). Collectively, these findings verify the successful synthesis of Fe-based SAN with atomically dispersed Fe centers in either Fe–N_4_ or Fe–SiN_3_ configurations.

**Fig. 3:**
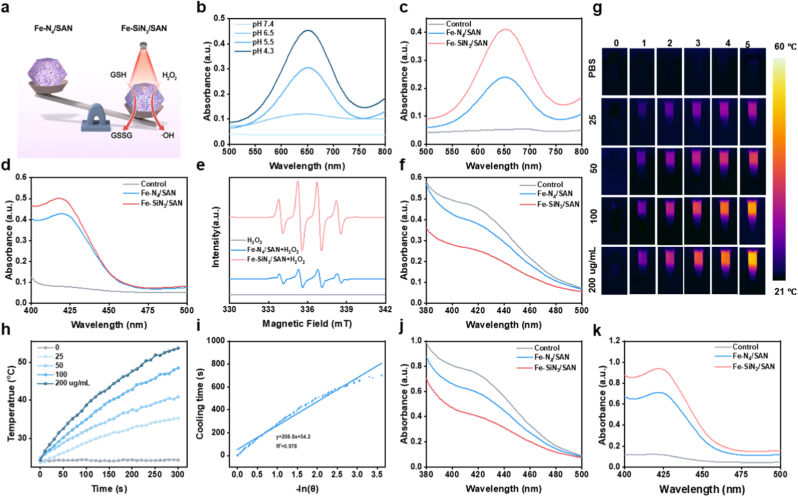
Photothermal-amplified catalytic activities of Fe–N_4_/SAN and Fe–SiN_3_/SAN. (a) Schematic representation of the catalytic properties of Fe–SiN_3_/SAN. (b) UV-vis spectra of TMB cultured with Fe–SiN_3_/SAN in the presence of H_2_O_2_ at various pH values. (c) UV-vis spectra of TMB and (d) ABTS cultured with Fe-based SANs in the presence of H_2_O_2_ at acidic pH. (e) ESR curves of •OH captured using a DMPO. (f) UV-vis spectra of DTNB cultured with varying formulations in the presence of GSH. (g) Infrared thermal (IT) imaging of various concentrations of Fe–SiN_3_/SAN. (h) Photothermal effect of Fe–SiN_3_/SAN following 808 nm laser irradiation. (i) Cooling curve of Fe–SiN_3_/SAN. (k) GSHOX- and (l) POD-like activities of Fe–SiN_3_/SAN following 808 nm laser irradiation.

Inspired by the catalytic potential conferred by polyvalent iron centers, we investigated the POD-mimicking activity and photothermal performance of the Fe-based SANs (Figure). POD-like activity was assessed using the chromogenic substrate 3,3′,5,5′-tetramethylbenzidine (TMB), which reflects •OH generation through changes in absorbance at 652 nm. As shown in Fig. 3a and b and S8, both Fe–N_4_/SAN and Fe–SiN_3_/SAN trigger a marked increase in TMB absorbance under mildly acidic conditions (pH 4.3–6.5) but display negligible catalytic activity at neutral pH (7.4). Moreover, their activity exhibits a concentration-dependent trend (Fig. S10). Notably, Fe–SiN_3_/SAN induced a more rapid and pronounced increase in absorbance than Fe–N_4_/SAN, indicating superior catalytic efficiency, likely attributable to the Si-mediated electronic modulation of the Fe active site (Fig. 3c). ABTS was used as an alternative probe to confirm the • OH generation. As shown in Fig. 3d and S11, Fe-SiN_3_/SAN exhibited a significantly greater enhancement in ABTS absorbance than Fe–N_4_/SAN in the presence of H_2_O_2_, further demonstrating the enhanced catalytic performance conferred by Si doping. Electron spin resonance (ESR) analysis was used to detect the • OH intermediate using 5,5-dimethyl-1-pyrroline N-oxide (DMPO) as a spin-trapping agent. As displayed in Fig. 3e, characteristic quartet peaks with an intensity ratio of 1:2:2:1 were observed, confirming that Fe-SiN_3_/SAN triggered higher levels of •OH production compared to Fe-N_4_/SAN. GSHOX-like activity was examined using the DTNB assay. An obvious decrease in absorbance at 412 nm was observed for both Fe-SiN_3_/SAN and Fe-N_4_/SAN, confirming effective GSH depletion (Fig. 3f and S13). Importantly, Fe–SiN_3_/SAN displayed a greater GSH-depletion capacity than Fe–N_4_/SAN, suggesting a stronger redox activity, consistent with its superior POD-like activity. In addition to its catalytic activity, Fe–SiN_3_/SAN exhibited notable photothermal conversion properties. Upon 808 nm laser irradiation, the temperature of the Fe–SiN_3_/SAN solutions rapidly increased within 5 min, whereas negligible temperature changes were observed in the PBS group (Fig. 3g and h). Furthermore, Fe–SiN_3_/SAN retained excellent thermal stability over four irradiation cycles, as evidenced by consistent temperature profiles (Fig. S14). The photothermal conversion efficiency (PCE) of Fe–SiN_3_/SAN was calculated to be 34.7% based on Roper's method (Fig. 3i), indicating its promising application in photothermal therapy (PTT). To explore the potential synergy between the catalytic and photothermal effects, the enzymatic activity of Fe–SiN_3_/SAN under 808 nm irradiation was evaluated. Post-irradiation DTNB and ABTS assays showed significantly enhanced GSH depletion and free radical generation, respectively (Fig. 3j and k), highlighting the enhancement of catalytic performance via photothermal activation. Collectively, these results demonstrate that Fe–SiN_3_/SAN exhibits superior POD- and GSHOX-like catalytic activities compared to Fe–N_4_/SAN and that these functions are further enhanced under NIR irradiation. This improved performance is attributed to Si doping at the Fe coordination site, which modulates the electronic structure and reactivity of the Fe center.

**Fig. 4:**
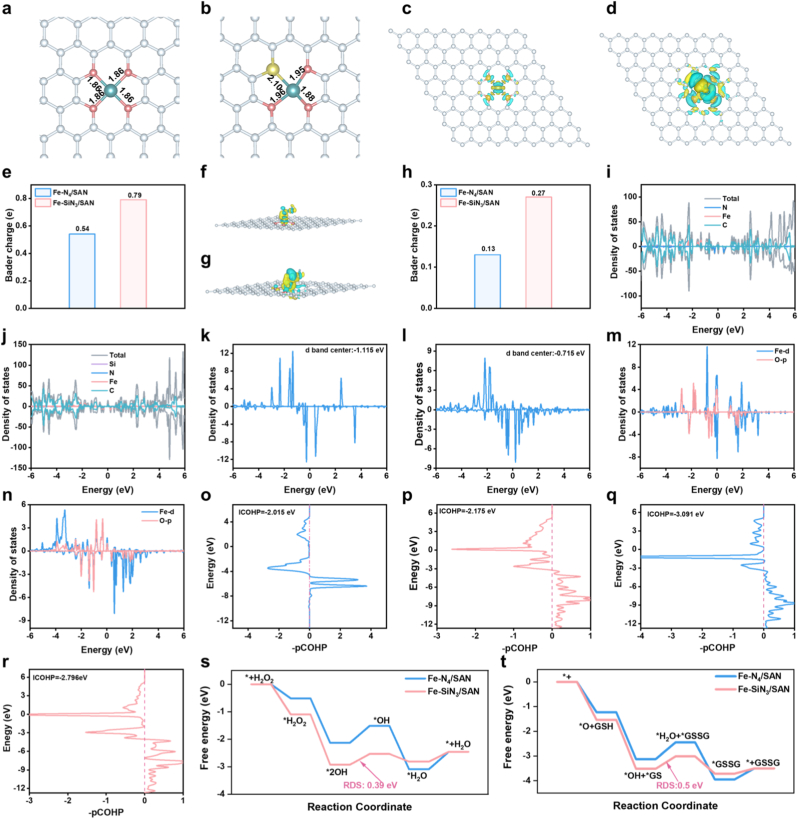
(a) Optimistic structures of symmetric Fe–N_4_/SAN and asymmetric Fe–SiN_3_/SAN. (c) The CDD of Fe–N_4_/SAN and (d) Fe–SiN_3_/SAN structures, with the isosurface value set at 0.01 e/Å^3^. The yellow area indicates electron accumulation, while the blue region represents electron depletion. (e) Bader charge of Fe–N_4_/SAN and (d) Fe–SiN_3_/SAN structures. (f) The CDD of Fe–N_4_/SAN and (g) Fe–SiN_3_/SAN structures upon adsorption of H_2_O_2_, with an isosurface value set at 0.005 e/Å^3^. (h) Bader charge of Fe–N_4_/SAN and Fe–SiN_3_/SAN structures upon H_2_O_2_ adsorption. (i) DOS analysis of Fe, C, N, and Si atoms in Fe–N_4_/SAN and (j) Fe–SiN_3_/SAN. (k) DOS analysis of Fe 3d band in Fe–N_4_/SAN and (l) Fe–SiN_3_/SAN. (m) DOS analysis of p-d orbital hybridization induced by symmetric Fe–N_4_/SAN (n) and asymmetric Fe–SiN_3_/SAN. (o) ICOHP of the bonds for the adsorption of O by Fe–N_4_/SAN and (p) Fe–SiN_3_/SAN. (q) The ICOHP of the bonds for the adsorption of GSSG by Fe–N_4_/SAN and (r) Fe–SiN_3_/SAN. (r) Gibbs free-energy diagrams for the decomposition of H_2_O_2_ into •OH and (s) the conversion of GSH into GSSG on Fe–N_4_/SAN and Fe–SiN_3_/SAN. (

To further elucidate the underlying mechanisms driving the enhanced catalytic performance induced by Si-doping, DFT calculations were conducted. Geometrically optimized structural diagrams of Fe–N_4_/SAN and Fe–SiN_3_/SAN are shown in Fig. S15. As depicted in Fig. 4a and b, Fe–N_4_/SAN exhibits a bond length of approximately 1.8 Å, which is indicative of its symmetrical and uniformly distributed architecture. In contrast, Fe–SiN_3_/SAN exhibited a notable elongation of the Fe–N bond, while the Fe–Si bond spans approximately 2.1 Å. Consequently, the system adopts an asymmetric and uneven structure, resulting in electron holes. This alteration results in an asymmetric and non-uniform structural configuration, which in turn generates electrons and holes. These electron holes play a pivotal role in significantly enhancing the adsorption of H_2_O_2_ and GSH, thereby catalyzing subsequent reactions and bolstering the overall catalytic efficacy of the system. The charge density difference (CDD) and Bader charge analyses of the Fe–N_4_/SAN and Fe–SiN_3_/SAN structures demonstrated that upon the incorporation of Si, Fe–SiN_3_/SAN exhibited a pronounced increase in electron transfer (Fig. 4c–e). Concurrently, the electronic activation capability of Fe–SiN_3_/SAN was significantly enhanced, thereby enhancing the overall catalytic performance. The CDD and Bader charge analyses of the Fe–N_4_/SAN and Fe–SiN_3_/SAN structures upon H_2_O_2_ adsorption revealed that the incorporation of Si significantly enhanced the adsorption capacity for H_2_O_2_ (Fig. 4f–h). This enhancement was accompanied by a marked increase in electron transfer and a corresponding increase in charge density, thereby boosting the catalytic activity of Fe–SiN_3_/SAN. Furthermore, density of states (DOS) analysis showed that Fe–SiN_3_/SAN exhibited a relatively continuous DOS profile compared to Fe–N_4_/SAN, which is highly beneficial for electron conduction as it amplifies the overall electrical conductivity (Fig. 4i and j). Additionally, the DOS analysis of the Fe 3d band revealed that the introduction of Si caused the d orbitals of Fe to shift closer to the Fermi level, thereby enhancing the adsorption capability for O and H_2_O_2_ (Fig. 4k and l). DOS analysis of p-d orbital hybridization shows a significant coupling effect between the d orbitals of Fe and the p orbitals of O. Importantly, the coupling strength of asymmetric Fe–SiN_3_/SAN is superior to symmetric Fe–N_4_/SAN (Fig. 4m and n), which enhances the adsorption of O as well as the adsorption and transformation of GSH. An integrated crystal orbital overlap population (ICOHP) analysis of the bonds for the adsorption of O revealed that a lower ICOHP value corresponds to a stronger bond energy.

For asymmetric Fe–SiN_3_/SAN, the incorporation of Si enhanced the energy of the Fe–O and Fe–S bonds with O and GSSG. This enhancement not only boosted the ability to adsorb and convert O but also weakened the adsorption of GSSG, thereby increasing its desorption (Fig. 4o–r). The pathways catalyzed by the Fe–SiN_3_/SAN and Fe–N_4_/SAN sites are shown in Figs. S16 and S17, respectively. Given that •OH was detected as a ROS during catalysis, the catalytic pathway can be delineated into five distinct steps: (1) adsorption of H_2_O_2_ on the catalyst surface (∗H_2_O_2_) and (2) homolytic cleavage of the O-O bond in H_2_O_2_, yielding two adsorbed OH groups on the catalytic sites (2OH). (3) (RDS) Release of •OH radical and formation of a single adsorbed ∗OH on the catalytic sites (∗OH + •OH). (4) The reduction of adsorbed ∗OH to form adsorbed ∗H_2_O at the catalytic sites (∗H_2_O). (5) Desorption of H_2_O from catalytic sites, thereby reactivating the catalyst (+H_2_O). The catalytic pathways and corresponding energy changes in an acidic solution (pH = 4) are shown in Fig. 4s. The RDS for the release of •OH radicals at the asymmetric Fe–SiN_3_ catalytic site exhibited an energy decrease (0.39 eV), which was significantly lower than the corresponding energy barrier (0.62 eV) at the Fe–N_4_ catalytic site. This lower energy barrier indicates that the Fe–SiN_3_ configuration more readily facilitates the release of •OH radicals. Similarly, the RDS in the conversion of GSH to GSSG was the formation of GSSG through the coupling of GS intermediates (Figs. S18 and S19). Asymmetric Fe–SiN_3_/SAN exhibits a lower energy barrier of 0.50 eV for this process, compared to the symmetric Fe–N_4_/SAN, which has an energy barrier of 0.67 eV. This lower energy barrier suggests that Fe–SiN_3_/SAN is more favorable for the formation and subsequent desorption of GSSG (Fig. 4t). These DFT calculations confirm that an asymmetric Fe–SiN_3_/SAN structure, achieved through Si-doping engineering of the symmetric Fe–N_4_/SAN, effectively enhanced the catalytic performance for the activation of H_2_O_2_ and GSH.

**Fig. 5:**
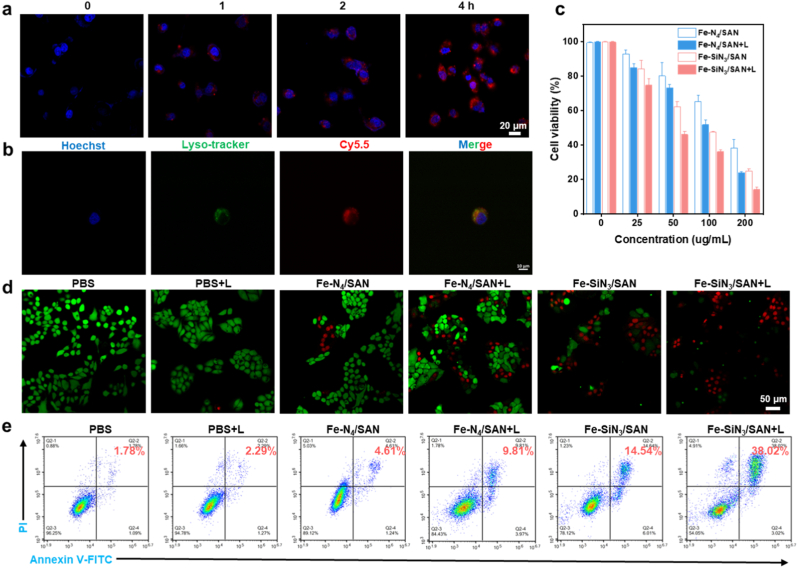
(a) CLSM images of Eca109 cells treated with Cy5.5-labeled Fe–SiN_3_/SAN. (b) CLSM images of Eca109 cell colocalization. (c) Tumor cell viability after 24 h of incubation with varying concentrations of Fe–SiN_3_/SAN upon laser irradiation. (d) Fluorescence images of live/dead staining for Eca109 cells incubated with varying formulations. (e) Flow cytometry measurements of Eca109 cells after incubation with varying formulations.

The cellular uptake and cytotoxic effects of the Fe-based SANs were evaluated using Eca109 cells. To investigate intracellular localization, Cy5.5-labeled Fe–SiN_3_/SAN was used. Confocal laser scanning microscopy (CLSM) revealed the time-dependent accumulation of red fluorescence within Eca109 cells, indicating progressive nanozyme internalization (Fig. 5a and S20). Further colocalization analysis showed that Fe–SiN_3_/SAN was primarily localized within lysosomes and the cytoplasm (Fig. 5b), which is consistent with the acidic microenvironment required to facilitate Fenton-like reactivity. Cell viability was assessed using the Cell Counting Kit-8 (CCK-8) assay. We have studied the anti-proliferation effect of Fe-SiN_3_/SAN on different normal cells (Fig. S21). The result demonstrated that Fe-SiN_3_/SAN had poor inhibition effect on cell growth, further confirming that Fe-SiN_3_/SAN exhibited superior catalytic performances under tumor microenvironment. We have provided quantitative data on cell survival rates in relation to nanozyme concentration and treatment time. As shown in Fig. S22, Fe-SiN_3_/SAN showed dose- and time-dependent effects on the inhibition of tumor growth. As the concentration increases and the time extends, the Fe-SiN_3_/SAN exhibits a higher anti-tumor effect. In addition, we conducted rescue experiments using the specific ferroptosis inhibitor ferrostatin-1 (Fer-1). Briefly, tumor cells were treated with Fer-1 (1 μM) for 24 h in the presence of Fe-N_4_/SAN or Fe-SiN_3_/SAN. Then cell viability was assessed (Fig. S23). As shown in Fig. 5c, both Fe–N_4_/SAN and Fe–SiN_3_/SAN exhibited dose-dependent cytotoxicity. Notably, Fe–SiN_3_/SAN demonstrates a more pronounced inhibitory effect than Fe–N_4_/SAN at all tested concentrations. Under laser irradiation (808 nm) and at a concentration of 200 μg/mL, Fe–SiN_3_/SAN reduces cell viability by 85.86%, significantly higher than the 63.87% inhibition observed in the absence of laser treatment, confirming the synergistic effect of photothermal enhancement. To further confirm cytotoxic effects, a live/dead co-staining assay was conducted using calcein-AM and propidium iodide (PI). As shown in Fig. 5d and S24, Fe–SiN_3_/SAN-treated cells exhibit stronger red fluorescence (PI) and reduced green fluorescence (calcein-AM) compared to the Fe–N_4_/SAN group, suggesting greater cell membrane compromise and higher levels of cell death. The anti-proliferative effect of Fe–SiN_3_/SAN was further amplified by laser irradiation. Additionally, the cell death rate was quantified using flow cytometry with Annexin V-FITC and propidium iodide (PI) double staining. The results demonstrated a significant increase in the proportion of cell death rates in the Fe–SiN_3_/SAN plus laser group compared with the other groups (Fig. 5e and S25), validating the enhanced therapeutic efficacy resulting from the combined catalytic and photothermal effects. These findings highlight the superior antiproliferative activity of Fe–SiN_3_/SAN relative to that of Fe–N_4_/SAN, which is attributed to its enhanced intracellular uptake, stronger redox catalytic activity, and photothermal synergy.

**Fig. 6:**
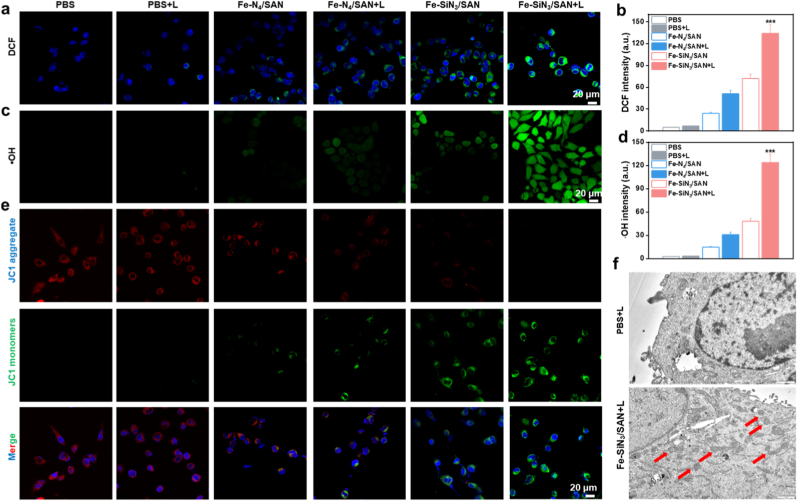
(a) DCF fluorescence images and (b) corresponding quantification of Eca109 cells following varying treatments. (c) Fluorescence images and (d) corresponding quantification of •OH probe O26-stained Eca109 cells following varying treatments. (e) JC-1 fluorescence images of Eca109 cells following varying treatments. (f) Bio-TEM of Eca109 cells following varying treatments. ∗∗∗P < 0.001.

To elucidate the mechanism underlying the antiproliferative effects on Eca109 cells, we investigated their influence on intracellular oxidative stress and ferroptosis pathways. Considering the POD-mimicking activity and photothermal properties of these nanozymes, the fluorescent probe DCFH-DA was used to assess intracellular ROS levels. As shown in Fig. 6a and b, and S26, CLSM revealed a significantly higher green fluorescence intensity in cells treated with Fe–SiN_3_/SAN than in cells treated with Fe–N_4_/SAN, indicating superior POD- and GSHOX-like catalytic ROS generation and GSH depletion. This effect was markedly enhanced by 808 nm laser irradiation. Furthermore, the • OH-specific probe O27 was used to detect •OH levels. Fig. 6c and d reveal significantly superior green fluorescence intensity within tumor cells incubated with Fe–SiN_3_/SAN than Fe–N_4_/SAN, indicating superior POD-like catalytic activity. To verify the specificity of •OH detection, validation experiments were performed using the •OH-specific fluorescent probe HPF. The results showed that both the HPF fluorescence signal were significantly enhanced (Fig. S27). Elevated ROS levels can trigger ferroptosis through disruption of mitochondrial membrane potential (MMP). To evaluate MMP changes, the mitochondrial probe JC-1 was used, which exhibited a fluorescence shift from red (aggregates) to green (monomers) upon mitochondrial depolarization. The CLSM images showed that both Fe–SiN_3_/SAN and Fe–N_4_/SAN induced MMP loss, with laser irradiation further amplifying the green fluorescence intensity, indicating severe mitochondrial dysfunction (Fig. 6e and S28). These observations were corroborated by biological transmission electron microscopy (Bio-TEM), which confirmed mitochondrial structural damage in cells treated with Fe–SiN_3_/SAN plus a laser, a hallmark feature of ferroptosis (Fig. 6f).

**Fig. 7:**
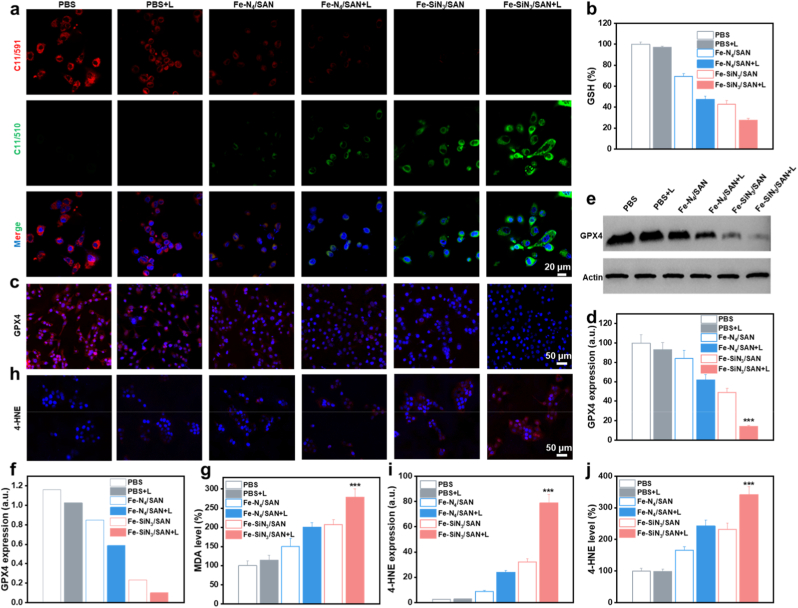
(a) Confocal images of fluorescent indicator C11-BODIPY^581/589^-stained Eca109 cells following varying treatments. (b) GSH levels in Eca109 cells following varying treatments. (c) Immunofluorescence images and (d) corresponding quantification of GPX4 expression in Eca109 cells following varying treatments. (e) WB images and (f) quantitative analysis of GPX4 expression in Eca109 cells following varying treatments. (g) MDA levels in Eca109 cells following varying treatments. (h) Immunofluorescence images and (i) quantitative analysis of 4-HNE expression in Eca109 cells following varying treatments. (j) 4-HNE levels in Eca109 cells using ELISA. ∗∗∗P < 0.001.

ROS-mediated LPO is central to ferroptosis; however, high intracellular glutathione (GSH) levels can counteract oxidative damage. The catalytic production of •OH, coupled with GSH depletion, drove irreversible LPO production, which was further monitored using the LPO-sensitive fluorescent probe C11-BODIPY^581/591^. The CLSM results (Fig. 7a and S29) revealed a significant increase in green fluorescence intensity accompanied by diminished red fluorescence in Fe–SiN_3_/SAN-treated cells, confirming robust LPO accumulation. This effect is further enhanced by laser irradiation. The measurement of intracellular GSH levels revealed significant depletion following treatment with Fe-based SANs (Fig. 7b). Notably, Fe–SiN_3_/SAN exhibits greater GSH consumption compared to Fe–N_4_/SAN, with laser irradiation further potentiating this effect, thereby amplifying cellular oxidative stress. Given that GSH depletion leads to GPX4 inactivation, an essential regulator of lipid repair and the inhibition of ferroptosis, we examined GPX4 expression. Both immunofluorescence staining and Western blot analyses exhibit a pronounced reduction in GPX4 protein levels in Fe–SiN_3_/SAN-treated cells compared to Fe–N_4_/SAN, consistent with enhanced catalytic activity facilitated by Si doping (Fig. 7c–f and S30). Consistent with these findings, malondialdehyde (MDA) and 4-HNE measurements demonstrated that Fe–SiN_3_/SAN induced the highest levels of MDA, 4-HNE, and classical LPO markers (Fig. 7g–j and S31). Collectively, these results indicated that Fe–SiN_3_/SAN effectively promoted ferroptosis in Eca109 cells by enhancing ROS generation, depleting GSH, inactivating GPX4, and inducing LPO, which further amplified these therapeutic effects.

**Fig. 8:**
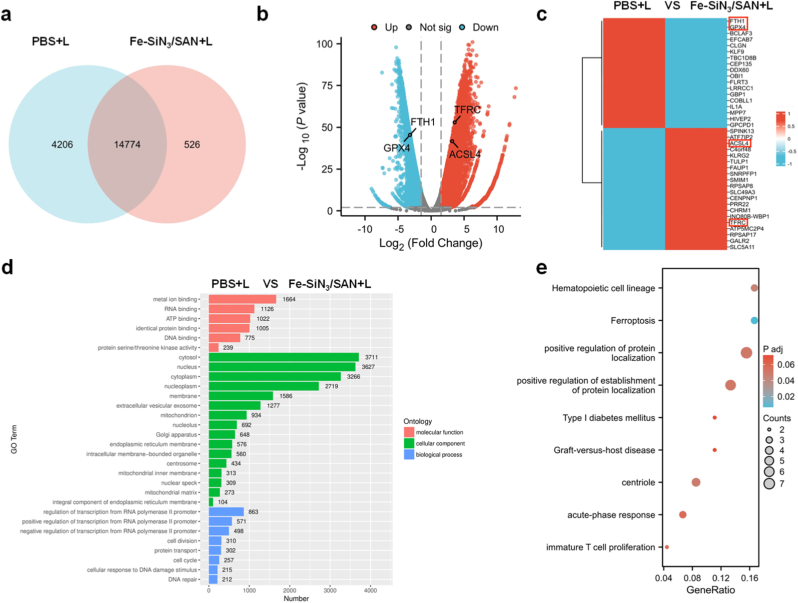
(a) Venn diagram comparing the number of differentially expressed genes between the two groups. (b) Volcano plot and (c) heatmap depicting differential gene expression between groups. (d) Functional enrichment plot of GO terms (e) KEGG pathway enrichment.

To analyze the transcriptomic differences between the control and Fe–SiN_3_/SAN plus laser groups in depth, we performed RNA sequencing data analysis on cells from both groups. The Venn diagram shows the overlap and specificity of differentially expressed genes (DEGs) across conditions. While 14,774 core DEGs were shared between the control and Fe–SiN_3_/SAN plus laser groups, the Fe–SiN_3_/SAN plus laser group exhibited 526 unique DEGs (Fig. 8a), suggesting both common and distinct transcriptomic responses to treatment. The volcano plot visually illustrates the genes that were significantly upregulated and downregulated between the groups (Fig. 8b). Among these, ferroptosis suppressor genes such as GPX4 and FTH1 were downregulated, whereas the ferroptosis inducer genes such as ACSL4 were upregulated in the treatment group. Similarly, the heatmap analysis results demonstrated that the genes were significantly upregulated and downregulated between the two groups (Fig. 8c). Additionally, gene ontology (GO) enrichment analysis revealed the distribution of DEGs across three categories (Fig. 8d): biological process (BP), molecular function (MF), and cellular components (CCs). KEGG pathway enrichment analysis suggested that pathways such as ferroptosis, centriole-related pathways, and positive regulation of protein localization may play critical roles in Fe–SiN_3_/SAN-induced Eca109 cell death (Fig. 8e).

**Fig. 9:**
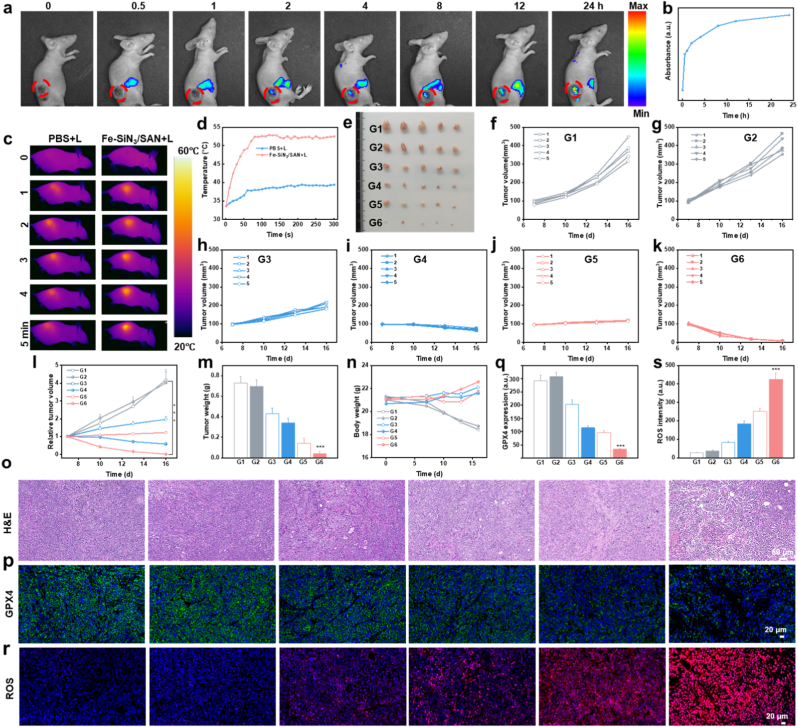
(a) Fluorescence images and (b) corresponding quantification of Eca109 tumor-bearing nude mice at various time points following the injection of Cy5.5-labeled Fe–SiN_3_/SAN. (c) Thermal images and (d) corresponding tumor temperature variations upon laser irradiation. (e) Tumor images collected from nude mice at the conclusion of the treatment. (f-k) Tumor growth curves of Eca109 tumor-bearing nude mice following varying treatments. (l) Relative tumor volume of nude mice following varying treatments. (m) Tumor weights collected from mice at the conclusion of the treatment. (n) Body weight curves of mice following varying treatments. (o) H&E staining of tumor slides harvested from varying groups. (p) Immunofluorescence and (q) corresponding quantification of GPX4 expression in tumor tissues following varying treatments. (r) Immunofluorescence and (s) corresponding quantification of ROS expression in tumor tissues following varying treatments. G1:PBS; G2:PBS + L; G3:Fe–N_4_/SAN; G4: Fe–N_4_/SAN + L; G5:Fe–SiN_3_/SAN; G6:Fe–SiN_3_/SAN + L. ∗∗∗P < 0.001.

Animal experiments were performed according to the protocol approved by the Ethical Committee of Fujian Medical University (IACUC FJMU2022-0608). Before evaluating the therapeutic efficacy of Fe–SiN_3_/SAN in vivo, its biocompatibility was systematically assessed. Hemolysis assays performed at various concentrations of Fe-SiN_3_/SAN (up to 200 μg/mL) demonstrate negligible hemolytic activity, indicating excellent biosafety (Fig. S32). Furthermore, hematoxylin and eosin (H&E) staining of the major organs revealed no discernible histopathological abnormalities, confirming the minimal systemic toxicity of the Fe-SiN_3_/SAN treatment (Fig. S33). The biodistribution of the Fe-SiN_3_/SAN was investigated using an IVIS imaging system following the intravenous administration of Cy5.5-labeled Fe-SiN_3_/SAN. Fluorescence imaging showed the progressive accumulation and retention of Fe-SiN_3_/SAN at tumor sites, with sustained high-intensity signals observed up to 24 h post-injection, likely attributable to the enhanced permeability and retention (EPR) effect (Fig. 9a and b, and S34). Concurrently, infrared thermal imaging revealed a rapid temperature increase at the tumor sites in Fe–SiN_3_/SAN-treated mice under 808 nm laser irradiation, whereas the control groups showed no significant temperature changes, confirming the strong photothermal conversion capability of Fe-SiN_3_/SAN (Fig. 9c and d).

To assess the antitumor efficacy, an Eca109 subcutaneous xenograft model was established. Tumor growth curves indicated that Fe–SiN_3_/SAN treatment significantly suppressed tumor progression compared to the controls, with notably stronger tumor inhibition than Fe–N_4_/SAN (Fig. 9e–l). Importantly, laser irradiation synergistically enhanced the tumor-suppressive effect of Fe–SiN_3_/SAN, consistent with photothermally enhanced catalytic therapy leading to improved therapeutic outcomes. At the study endpoint, the average tumor weight in the Fe–SiN_3_/SAN plus laser group was markedly lower than those in the other groups, demonstrating the highest tumor growth inhibition rate (Fig. 9m). Throughout the treatment period, no significant body weight loss was observed in any groups, indicating favorable in vivo biocompatibility (Fig. 9n). Histological examination of the excised tumors revealed pronounced ferroptotic features and reduced cellular proliferation in the Fe–SiN_3_/SAN plus laser group, as evidenced by H&E and TUNEL staining (Fig. 9o and S35). The IHC results showed that the expression of 4-HNE in the tumors of the Fe–SiN_3_/SAN plus laser group increased (Fig. S36). Immunofluorescence analysis corroborated these findings, showing a significant downregulation of GPX4 expression following combined Fe–SiN_3_/SAN and laser treatment (Fig. 9p and q and S37). The ROS staining results showed that the ROS levels increased in the Fe–SiN_3_/SAN plus laser group (Fig. 9s and S38). These results demonstrate that Fe–SiN_3_/SAN, particularly when combined with laser irradiation, effectively induces tumor ferroptosis by promoting LPO and GPX4 inactivation, underscoring its promising potential for cancer therapy.

## 3 Conclusion

Two distinct coordination environments were successfully fabricated: symmetric coordination with isolated Fe–N_4_ active sites and asymmetric coordination with isolated Fe–SiN_3_ active sites. The incorporation of Si induces substantial geometric distortion and electronic structure modulation via Fe–N bond stretching, effectively balancing the adsorption and desorption of oxygen intermediates and markedly strengthening the catalytic activity. Asymmetric Fe–SiN_3_/SAN demonstrated higher POD- and GSHOx-mimicking activities than its asymmetric Fe–N_4_/SAN counterparts, highlighting that Si doping enhances the transfer of electrons and optimizes the adsorption energy of the reaction intermediates. DFT calculations revealed that the asymmetric Fe–SiN_3_/SAN configuration enhanced the electron-activating capacity around the Fe center, increased the number of unpaired electrons, and shifted the Fe d-band center toward the Fermi level. This promoted H_2_O_2_ and GSH adsorption, accelerating the catalytic RDS. Notably, Bader charge analysis showed that asymmetric Fe–SiN_3_/SAN exhibited a lower desorption energy for the RDS intermediates ∗OH and ∗GS than symmetric Fe–N_4_/SAN, indicating its superior ROS-generating capability. The experimental results indicated that Fe–SiN_3_/SAN efficiently triggered irreversible tumor ferroptosis via the accumulation of LPO and inactivation of GPX4. Moreover, 808 nm laser irradiation further boosted the catalytic activity of the nanozyme, leading to significant tumor growth inhibition. This study presents a conspicuous method for regulating the coordination environments of SANs to amplify enzymatic activity, offering a promising avenue for biomedical applications.

## Ethics approval and consent to participate

Animal experiments were performed according to the protocol approved by The Ethical Committee of Fujian Medical University (IACUC FJMU2022-0608).

## Consent for publication

All authors agreed to submit this manuscript.

## Funding declaration

This work was supported by 10.13039/501100001809National Natural Science Foundation of China (32501233), 10.13039/501100003392Natural Science Foundation of Fujian Province (2025J01087), 10.13039/501100017686Fujian Provincial Health Technology Project (NO.2024CXA017), Fujian Provincial Health Commission's Young and Middle-aged Backbone Talent Project (NO.2024GGB08), Joint Funds for the Innovation of Science and Technology, Fujian Province (No.202519150).

## CRediT authorship contribution statement

**Bo Liu:** Conceptualization, Writing – original draft, Writing – review & editing. **Huihan Yi:** Conceptualization. **Zhan Zhuang:** Data curation. **Penghui Wei:** Formal analysis. **Hongjia Zheng:** Data curation, Formal analysis. **Huimin Wang:** Data curation, Formal analysis. **Yifei Tu:** Formal analysis. **Jinbao Xie:** Investigation. **Yang Zhu:** Supervision, Writing – original draft, Writing – review & editing. **Xu Li:** Supervision, Writing – original draft, Writing – review & editing.

## Declaration of competing interest

The authors declared that they have no known competing financial interests or personal relationships that could have appeared to influence the work reported in this paper.

## Data Availability

Data will be made available on request.
