# Supplementary material for: Silicon doping-engineered asymmetric coordination and electronic structure modulation for amplified single-atom nanozyme catalytic therapy
Source: Mater Today Bio. 2026 Apr 3;38:103082. doi: 10.1016/j.mtbio.2026.103082 (PMC13090732; doi:10.1016/j.mtbio.2026.103082)
Supplement: Multimedia component 1 [file mmc1.docx]

**Materials**

**Chemicals**

2,2’-azino-bis(3-ethylbenzothiazoline-6-sulfonic acid) diammonium salt (ABTS), I

ron cetylacetonate (Fe(acac)3), hydrogen peroxide (H_2_O_2_), acetic acid (HAc), sodium acetate (NaAc), and ethanol were purchased from Sinopharm Chemical Reagents (Shanghai, China). 3,3',5,5'-tetramethylbenzidine (TMB), and C11-BODIPY581/591 were provided by Sigma-Aldrich (St. Louis, USA). Hoechst 33342, 2′,7′-dichlorofluorescin diacetate (DCFH-DA), cell count kit-8 (CCK-8), annexin V-FITC/PI apoptosis detection kit, thiobarbituric acid (TBA), AM/PI, and 1,1',3,3'-tetraethyl-5,5',6,6'-tetrachloroimidacarbocyanine iodide (JC-1) were bought from Beyotime (Shanghai, China). Dulbecco's modified eagle medium (DMEM) was purchased from Hyclone (Logan, USA). 5,5-dimethyl-1-pyrroline N-oxide (DMPO) was bought from Dojindo (Dojindo). Cyanine 5.5 monosuccinimidyl ester (Cy5.5-NHS), ELISA, and annexin V-FITC/PI apoptosis detection kit were purchased from Beijing Solarbio Science & Technology Co., Ltd. (Beijing, China). Live & Dead Bacterial Staining Kit (Cat#40274ES60) and GMyc-PCR Mycoplasma Test Kit (Cat#40601) was purchased from Yeasen Biotechnology (Shanghai) Co., Ltd.. Deionized (DI) water was obtained from a Milli-Q water purification system.

**Instruments**

Powder X-ray diffraction (XRD) patterns were recorded on a Rigaku Miniflex-600 diffractometer. Transmission electron microscope (TEM) images were taken by Hitachi-7700. High-angle annular dark field scanning transmission electron microscopy (HAADF-STEM) images were recorded by JEM-ARM200F (JEOL) TEM/STEM with a spherical aberration corrector. The energy-dispersive X-ray spectroscopy (EDS) mapping was performed by JEM-2100F. X-ray photoelectron spectroscopy (XPS) spectra were collected on scanning X-ray microprobe (PHI 5000 Verasa, ULAC-PHI). Scanning electron microscopy (SEM) images were taken by Nova NanoSEM 230. Fluorescence imaging was performed by confocal microscopy (Nikon C2). The absorption spectra were measured by an ultraviolet-visible (UV-vis) UH4150 spectrophotometer (Hitachi). Metal content was measured by using inductively coupled plasma mass spectrometer (ICP-MS, PlasmaQuad 3, Thermo Elemental). Hydrodynamic diameters and zeta potentials were determined by a Zetasizer nano ZS instrument (Malvern). Cancer cell apoptosis was monitored by using a flow cytometer (CytoFLEX, Beckman).

**Methods**

**2.1. Synthesis of Fe-N_4_/SAN and Fe-SiN_3_/SAN**

Fe@ZIF-8 precursors were synthesized using a host-guest strategy. 3.54 g 2-methylimidazole and 100 mg Fe(acac)_3_ were added into a 150 mL flask containing 60 mL methanol as solution A. 1.6 g Zn(NO_3_)_2_•6H_2_O was dissolved in 60 mL methanol as solution B. Then solution A was mixed with solution B, and the mixture was further stirred for 12 h. Then the Fe@ZIF-8 powders were collected by centrifugation, washed with methanol several times, and dried at 65 °C in a vacuum oven overnight.

And the Fe-SiN_3_/SAN was obtained by the two-step pyrolysis strategy. Firstly, the Fe@ZIF-8 powder was placed successively in a tubular furnace, and then pyrolysis at 950 ºC for 3 h in an argon atmosphere, followed by 540 ºC for 3 h in an SiH_4_ atmosphere. The Fe-N_4_/SAN was prepared by similar procedures except for SiH_4_ species.

**2.2. Characterization of Fe-N_4_/SAN and Fe-SiN_3_/SAN**

The peroxidase (POD)-like catalytic activities of Fe-N_4_/SAN and Fe-SiN_3_/SAN were systematically evaluated using the 3,3′,5,5′-tetramethylbenzidine (TMB) colorimetric assay. In the presence of hydrogen peroxide (H_2_O_2_), both nanozymes catalyzed the oxidation of TMB to its blue-colored oxidized form (oxTMB), which exhibits a characteristic absorption peak at 652 nm. Specifically, TMB was added to individual solutions of Fe-N_4_/SAN and Fe-SiN_3_/SAN, followed by pH adjustment and incubation at room temperature for 20 minutes. After centrifugation, the absorbance of the resulting supernatants was measured at 652 nm. To assess the pH dependence of the catalytic activity, the TMB oxidation kinetics were further measured across a range of pH values. The glutathione (GSH)-depleting capacities of Fe-N_4_/SAN and Fe-SiN_3_/SAN were determined using the 5,5′-dithiobis(2-nitrobenzoic acid) (DTNB) assay. Each sample was incubated with DTNB in the dark at room temperature for 10 minutes, followed by centrifugation, and the absorbance of the supernatants was recorded at 412 nm. Additionally, the catalytic properties were further assessed using the 2,2′-azino-bis(3-ethylbenzothiazoline-6-sulfonic acid) (ABTS) assay. ABTS was mixed with either Fe-N_4_/SAN or Fe-SiN_3_/SAN, adjusted to the appropriate pH, and incubated at room temperature for 10 minutes before centrifugation. The absorbance of the supernatants was measured at 405 nm to evaluate radical scavenging activity. To investigate laser-responsive catalytic behavior, both ABTS and DTNB assays were repeated after 808 nm near-infrared (NIR) laser irradiation. The photothermal performance of Fe-N_4_/SAN and Fe-SiN_3_/SAN was evaluated by exposing the samples to 808 nm laser irradiation at room temperature, during which their temperature rise profiles were recorded to assess photothermal conversion efficiency. After reaching a stable maximum temperature, the samples were allowed to naturally cool to room temperature. This heating-cooling cycle was repeated four times, and the temperature changes during each cycle were monitored to evaluate the photothermal stability and reproducibility of the materials.

**2.2. Cellular Uptake and Lysosomal Colocalization of Cy5.5-Labeled Fe-SiN_3_/SAN**

Eca109 cells were seeded in glass-bottom culture dishes at a density of 1×10⁵ cells per dish and incubated overnight. Cells were then treated with Cy5.5-labeled Fe-SiN_3_/SAN (200 μg/mL) for 0.5, 1, 2, and 4 hours. Following treatment, cells were stained with Hoechst 33342 and LysoTracker Green for 20 minutes in the dark to label nuclei and lysosomes, respectively. After washing with phosphate-buffered saline (PBS), fluorescence images were captured by Zeiss LSM 780 confocal laser scanning microscope (CLSM). Images were acquired from randomly selected fields to analyze cellular uptake dynamics and the extent of lysosomal colocalization of the nanozyme over time.

**2.3. Cytotoxicity and Apoptosis Evaluation**

To evaluate the cytotoxic effects of Fe-N_4_/SAN and Fe-SiN_3_/SAN, Eca109 cells were seeded into 96-well plates at a density of 3×10³ cells per well and allowed to adhere for 12 hours. Cells were then treated with varying concentrations of Fe-N_4_/SAN or Fe-SiN_3_/SAN (0, 25, 50, 100, 200 μg/mL), followed by a 12-hour incubation. Depending on the experimental group, laser irradiation (808 nm) was subsequently applied. The experimental groups included: (1) control, (2) laser only, (3) Fe-N_4_/SAN only, (4) Fe-N_4_/SAN+laser, (5) Fe-SiN_3_/SAN only, and (6) Fe-SiN_3_/SAN+laser. After treatment, the medium was replaced with fresh culture medium containing 10 μL of Cell Counting Kit-8 (CCK-8) reagent. Following a 2-hour incubation at 37 °C, absorbance was measured at 450 nm using a microplate reader to assess cell viability. For fluorescence-based live/dead cell imaging, Eca109 cells were cultured in glass-bottom confocal dishes and incubated for 12 hours. Cells were then subjected to the same six treatment conditions. After treatment, cells were washed with PBS and stained with fluorescein diacetate (FDA, 5 μM) and propidium iodide (PI, 20 μM) for 20 minutes at 37 °C in the dark. The stained cells were imaged by CLSM to visualize cell viability and apoptosis. To further evaluate apoptosis, an Annexin V-FITC/PI apoptosis detection kit was employed. After appropriate treatment and staining according to the manufacturer’s protocol, cells were analyzed by flow cytometry to quantify apoptotic and necrotic populations.

**2.4. In Vitro Reactive Oxygen Species (ROS) Generation**

Intracellular ROS levels in the six experimental groups were quantified using the 2′,7′-dichlorofluorescin diacetate (DCFH-DA) fluorescent probe. Eca109 cells were seeded in glass-bottom confocal dishes and incubated with 10 μM DCFH-DA and Hoechst 33342 for 30 minutes at 37 °C in the dark. After incubation, excess probe was removed by washing with PBS. The resulting fluorescence intensity, reflecting intracellular ROS production, was visualized using confocal laser scanning microscopy (CLSM) with excitation at 488 nm and emission at 525 nm.

**2.5. Hydroxyl Radical (•OH) Generation Mediated by Fe-N_4_/SAN and Fe-SiN_3_/SAN**

The generation of hydroxyl radicals (•OH) was evaluated using the •OH-specific fluorescent probe O27. This membrane-permeable probe is selectively oxidized by intracellular hydroxyl radicals, emitting green fluorescence (~525 nm). Cells from the six experimental groups were incubated with O27 solution in the dark at room temperature for 30 minutes. After incubation, cells were washed three times with PBS to remove unbound probe. Fluorescent images were acquired by CLSM to assess •OH production.

**2.6. Assessment of Mitochondrial Membrane Potential (MMP) Changes**

Mitochondrial membrane potential (MMP) changes were analyzed using the 5,5′,6,6′-Tetrachloro-1,1′,3,3′-tetraethyl-imidacarbocyanine iodide (JC-1) dye assay. JC-1 is a lipophilic, cationic dye that selectively enters mitochondria. In healthy cells with high MMP, JC-1 aggregates within the mitochondrial matrix, emitting red fluorescence (~590 nm). In contrast, in cells with depolarized mitochondria, JC-1 remains in monomeric form and emits green fluorescence (~529 nm). Eca109 cells were seeded in confocal dishes and incubated with JC-1 and Hoechst 33342 staining solution at 37 °C for 30 minutes in the dark. After washing with PBS, fluorescence signals were captured by CLSM.

**2.7. Intracellular Structural Alterations**

The culture medium was removed, and cells were fixed with 2.5% glutaraldehyde at room temperature for 5 minutes. Cells were then gently scraped using a sterile cell scraper and centrifuged at 3000 rpm for 2 minutes. After discarding the supernatant, fresh electron microscopy fixative was added, and samples were incubated at room temperature in the dark for 30 minutes. The specimens were subsequently dehydrated, infiltrated, embedded, sectioned, and stained according to standard TEM protocols. Cellular ultrastructure was observed and imaged using a transmission electron microscope.

**2.8. In Vitro Lipid Peroxide (LPO) Detection**

LPO levels in the six experimental groups were assessed using the fluorescent probe BODIPY™ 581/591 C11. In its reduced form, BODIPY 581/591 C11 emits red fluorescence (~591 nm), whereas oxidation by LPO shifts the emission to green (~510 nm). Eca109 cells were seeded in glass-bottom confocal dishes and incubated with 10 μM BODIPY™ 581/591 C11 and Hoechst 33342. Following a 30-minute incubation at 37 °C in the dark, excess dye was removed by washing three times with PBS. Changes in fluorescence were monitored and imaged by CLSM.

**2.9. Immunofluorescence Analysis of GPX4 and 4-HNE**

To evaluate oxidative stress and ferroptosis-related protein expression, immunofluorescence staining for glutathione peroxidase 4 (GPX4) and 4-hydroxynonenal (4-HNE) was performed. Eca109 cells were seeded, subjected to the respective treatments for each group, and then fixed and permeabilized. After blocking, cells were incubated with primary antibodies against GPX4 and 4-HNE, followed by fluorescently labeled secondary antibodies. Nuclear counterstaining was performed with Hoechst 33342. CLSM was used for fluorescence imaging and quantification.

**2.10. GPX4 Protein Expression Analysis**

After removing the culture medium from all six groups, cells were washed with PBS and the residual liquid was completely aspirated. Each dish was then lysed with 100 μL of RIPA buffer containing protease inhibitors. The cells were scraped, transferred to Eppendorf tubes, and lysed on ice for 20 minutes at 4 °C. The lysates were centrifuged at 12,000 rcf for 10 minutes, and the supernatants were collected. Proteins were separated by SDS-PAGE and transferred onto PVDF membranes. After blocking, the membranes were incubated overnight at 4 °C with a primary antibody against GPX4, using β-actin as a loading control. The next day, the membranes were washed three times with TBST (10 minutes each), incubated with an appropriate HRP-conjugated secondary antibody, and developed using enhanced chemiluminescence (ECL). The intensity of the protein bands was quantified by densitometric analysis.

**2.11 In Vitro Measurement of Intracellular Glutathione (GSH) and Malondialdehyde (MDA) Levels**

Intracellular levels of glutathione (GSH) and malondialdehyde (MDA) were quantified using standard enzymatic assays. After lysis of cells from the six experimental groups, the resulting lysates were collected for analysis. GSH levels were measured using a Glutathione Assay Kit according to the manufacturer’s instructions, and absorbance was recorded at 405 nm using a spectrophotometer. MDA levels were determined with an MDA Assay Kit based on the formation of a thiobarbituric acid (TBA)-MDA adduct, whose absorbance was measured at 532 nm. The concentrations of GSH and MDA were calculated by referencing standard curves, following the kit protocols.

**2.12. RNA Sequencing Analysis of Ferroptosis-Related Gene Expression**

To investigate ferroptosis-related transcriptional changes, Eca109 cells in the control group and Fe-SiN_3_/SAN + laser group were treated with PBS, 200 μg/mL 200 μg/mL Fe-SiN_3_/SAN respectively, followed by continuous incubation for 12 hours. Subsequently, 808 nm laser irradiation was applied. After the treatment was completed, the cells were collected for RNA sequencing (RNA-seq). Total RNA was extracted using a standard RNA isolation protocol. RNA quality and quantity were assessed prior to sequencing. Transcriptomic profiling was performed to identify differentially expressed genes associated with ferroptosis pathways, and the expression patterns were analyzed to elucidate the regulatory mechanisms underlying nanozyme-induced ferroptosis.

**2.13 Hemolysis Assay**

Red blood cells were incubated with deionized water (positive control), PBS (negative control), and various concentrations of Fe-SiN_3_/SAN (200, 100, 50, and 25 μg/mL). The hemolysis rate (%) was calculated using the following formula: Hemolysis rate (%) = [(OD_sample − OD_negative) / (OD_positive − OD_negative)] × 100%, where the optical density (OD) was measured at 540 nm.

**2.14. In Vivo Thermal Imaging**

To evaluate the photothermal conversion performance of Fe-SiN_3_/SAN in vivo, Eca109 tumor-bearing nude mice were intravenously injected with a predetermined dose of Fe-SiN_3_/SAN. After 12 hours of systemic circulation, the tumor sites were irradiated with an 808 nm near-infrared (NIR) laser. A thermal imaging camera was used to monitor temperature changes in real time at the tumor site. The temperature variation was recorded and plotted to assess the photothermal heating effect.

**2.15 Animal Grouping and Treatment**

All animal procedures were approved by the Institutional Animal Care and Use Committee of Fujian Medical University. Male BALB/c nude mice (6-8 weeks old, 20 ± 2 g) were used to establish subcutaneous tumor xenograft models. After tumors reached an appropriate size, mice received intravenous injections of treatment agents via the tail vein. Mice in the control group were injected with an equal volume of saline. Treatments were administered every three days (150 μL, 150 μg/μL), for a total of three doses. Twelve hours after the first injection, laser irradiation was applied to the designated groups.

Mice were randomly divided into six groups (n = 5 per group):

(1) Control group: intravenous injection of saline.

(2) Laser group: laser irradiation (808 nm, 10 min) at the tumor site without treatment.

(3) Fe-N_4_/SAN group: intravenous injection of Fe-N_4_/SAN.

(4) Fe-N_4_/SAN + Laser group: intravenous injection of Fe-N_4_/SAN followed by 10 min of laser irradiation.

(5) Fe-SiN_3_/SAN group: intravenous injection of Fe-SiN_3_/SAN.

(6) Fe-SiN_3_/SAN + Laser group: intravenous injection of Fe-SiN_3_/SAN followed by 10 min of laser irradiation.

Tumor volume and body weight were monitored every three days. At the end of the experiment, tumors and major organs (heart, liver, spleen, lungs, kidneys) were harvested for histological and immunohistochemical analyses, including TUNEL staining, H&E staining, 4-HNE immunohistochemistry, and immunofluorescence staining for GPX4 and ROS.

**2.16 Histological and Immunofluorescence Staining**

Tumor tissues and major organs (liver, heart, spleen, lungs, and kidneys) were sectioned and subjected to hematoxylin and eosin (H&E) staining to evaluate histopathological features under a light microscope. Tumor sections were further analyzed by immunofluorescence and Immunohistochemistry staining to detect glutathione peroxidase 4 (GPX4), and 4-hydroxynonenal (4-HNE). After blocking, sections were incubated with primary antibodies specific to each target protein, followed by incubation with appropriate fluorescently labeled secondary antibodies. In addition, TUNEL staining was performed on tumor sections to assess apoptotic cell death in each group. ROS staining was performed to detect the level of ROS in tissues. Fluorescence signals were visualized and imaged using confocal laser scanning microscopy.

**2.17. In Vivo Fluorescence Imaging of Biodistribution**

To assess the biodistribution and tumor-targeting efficiency of Fe-SiN_3_/SAN, an in vivo imaging system (IVIS) was used to monitor Cy5.5-labeled nanozyme accumulation in tumor-bearing mice. Cy5.5-labeled Fe-SiN_3_/SAN was administered via tail vein injection, and whole-body fluorescence imaging was performed at predetermined time points (0, 0.5, 1, 2, 4, 8, 12, and 24 h post-injection). Mice were anesthetized using 2% isoflurane in oxygen and placed in the imaging chamber during image acquisition. At 24 hours post-injection, major organs (heart, liver, spleen, lungs, kidneys, and tumor) were harvested for ex vivo fluorescence imaging to further evaluate the biodistribution of Fe-SiN_3_/SAN.

**
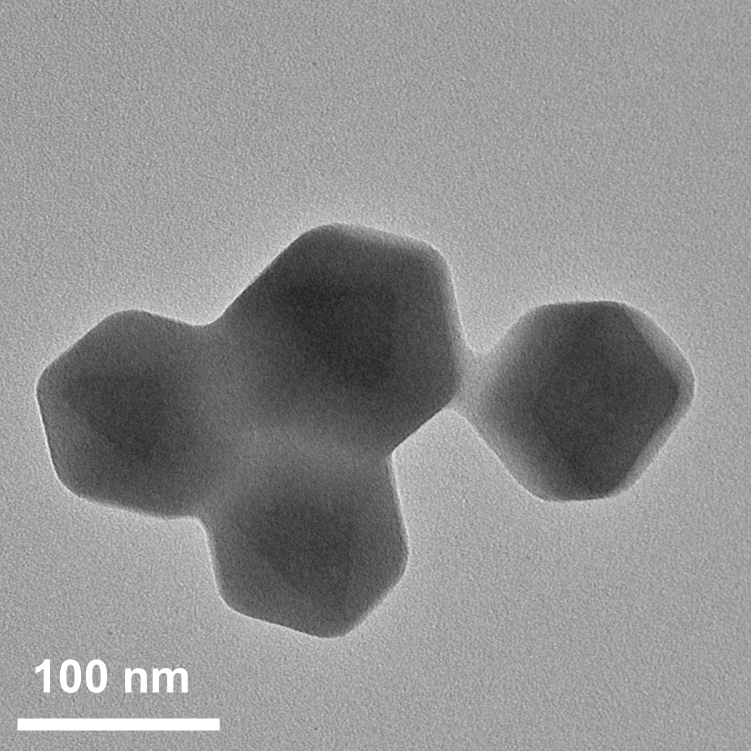
**

**Figure S1.** TEM of Fe@ZIF-8.

**
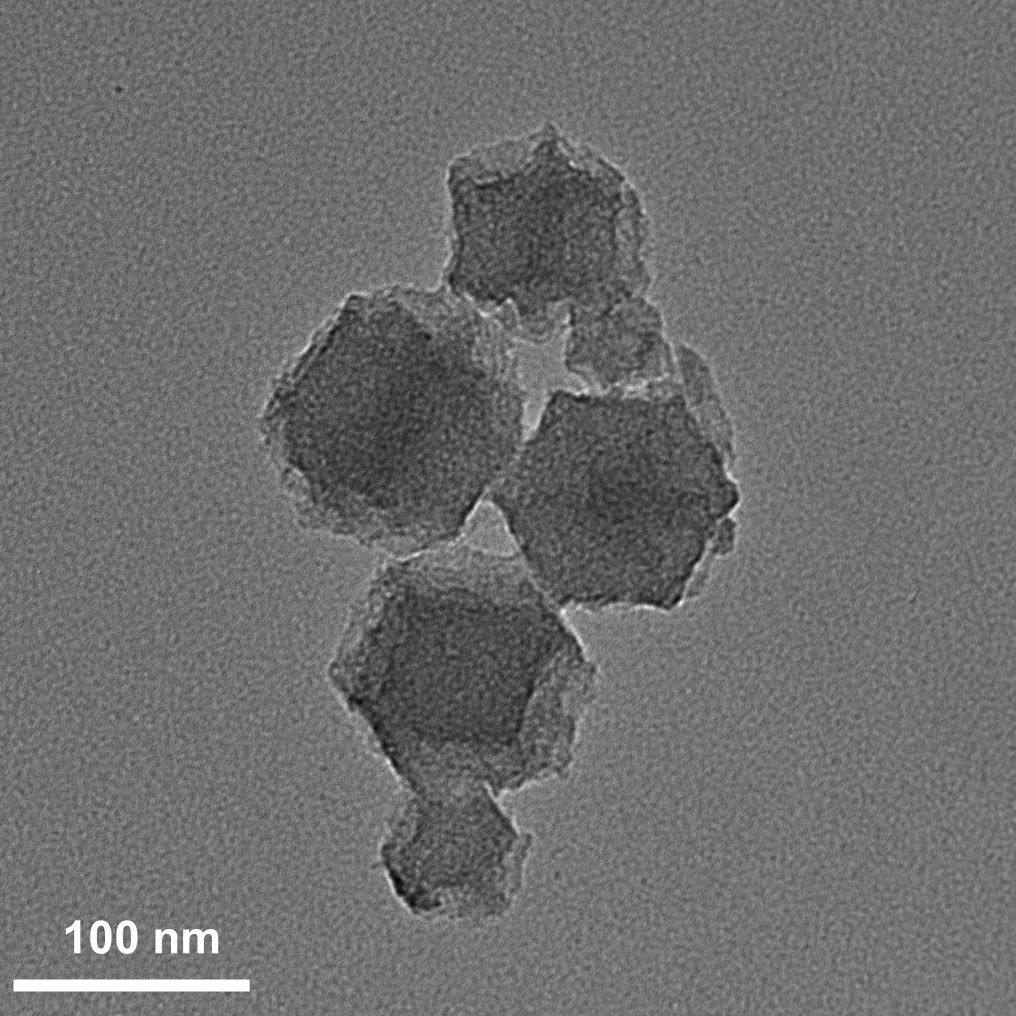
**

**Figure S2.** TEM of Fe-N_4_/SAN.

**
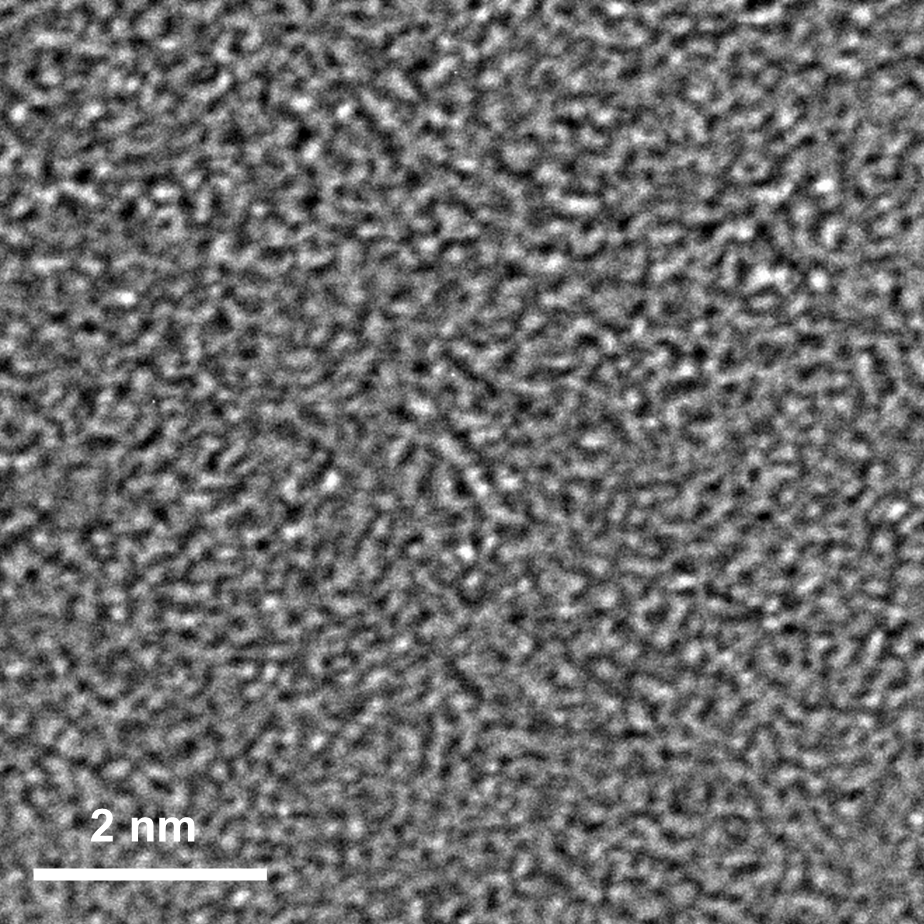
**

**Figure S3.** HR-TEM of Fe-N_4_/SAN.


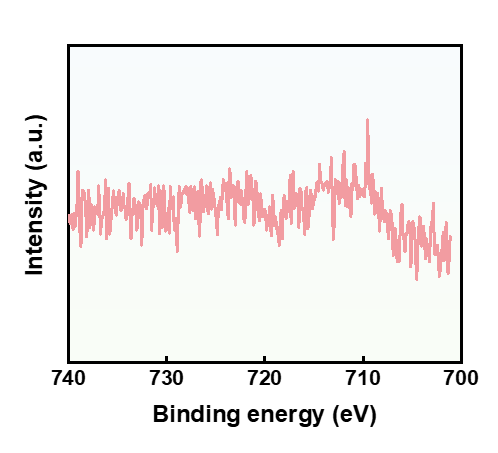


**Figure S4.** Fe 2p XPS in Fe-BrN_3_/SAN.


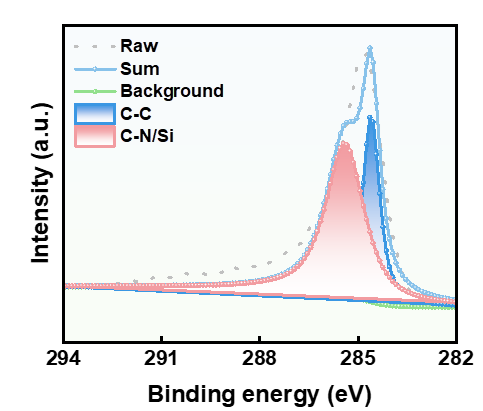


**Figure S5.** C 1s XPS in Fe-BrN_3_/SAN.


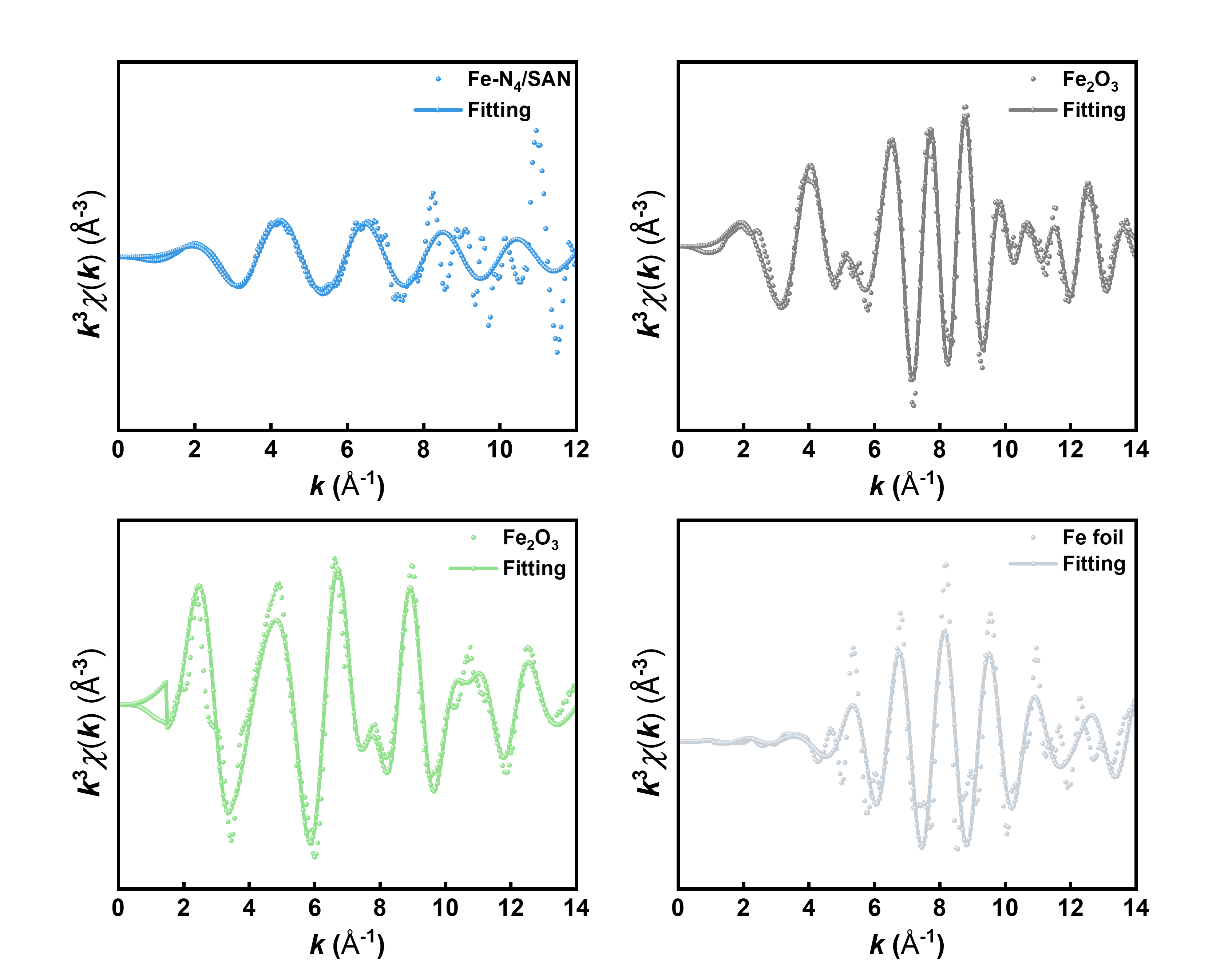


**Figure S6.** EXAFS fitting curves of different materials at the k space.

**Table S1. EXAFS data fitting results of Samples.**


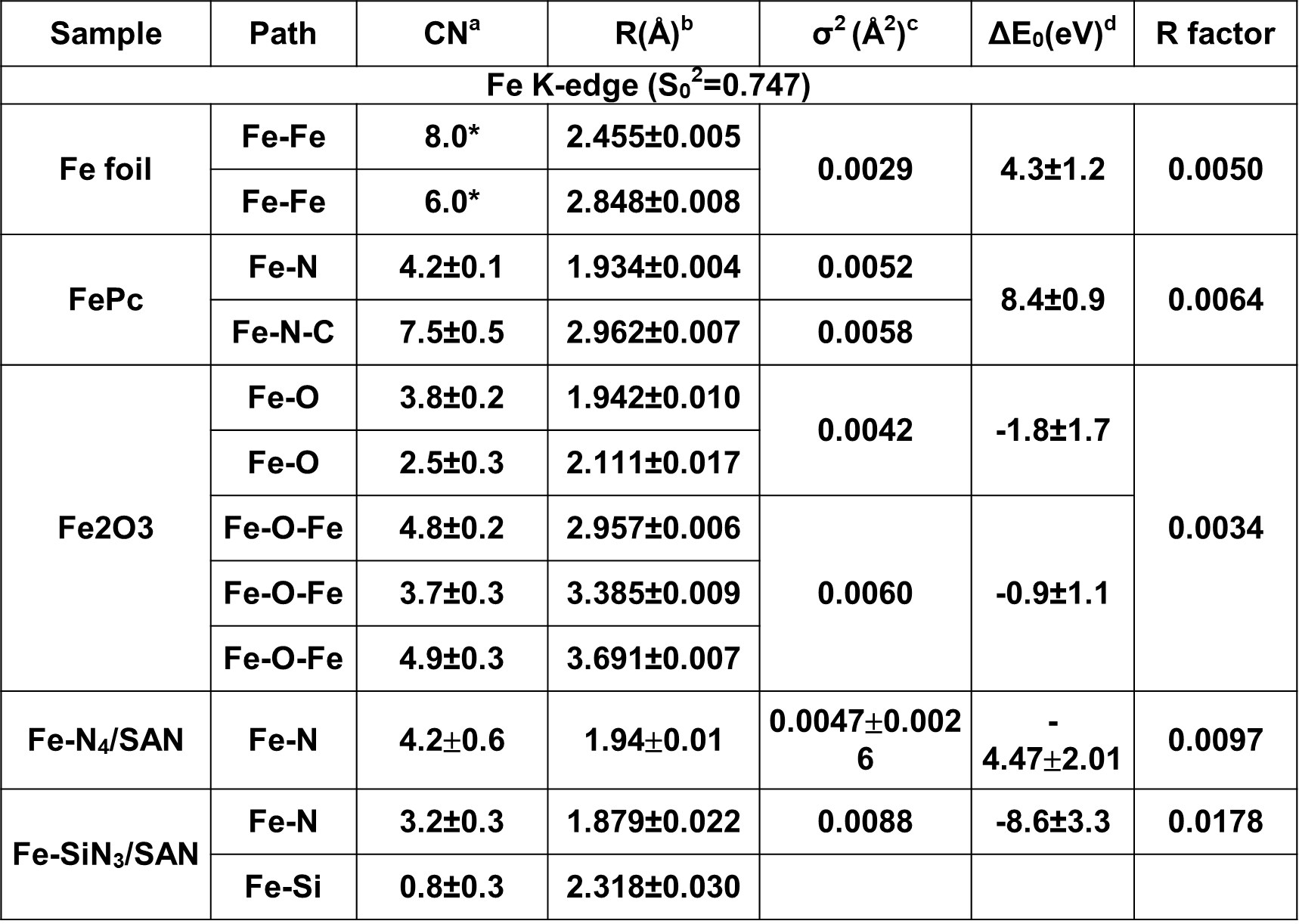


*^a^CN*, coordination number; *^b^R*, the distance between absorber and backscatter atoms; *^c^σ*^2^, the Debye Waller factor value; *^d^ΔE*_0_, inner potential correction to account for the difference in the inner potential between the sample and the reference compound; *R* factor indicates the goodness of the fit. *S*_0_^2^ was fixed to 0.747, according to the experimental EXAFS fit of Fe foil by fixing *CN* as the known crystallographic value. * This value was fixed during EXAFS fitting, based on the known structure of Fe. Fitting conditions: *k* range：2.0 - 9.0; *R* range: 1.0-2.5; fitting space: R space; *k*-weight = 3. A reasonable range of EXAFS fitting parameters: 0.700 < *Ѕ*_0_^2^ < 1.000; *CN >* 0; *σ*^2^ > 0 Å^2^; |Δ*E*_0_| < 15 eV; *R* factor < 0.02.


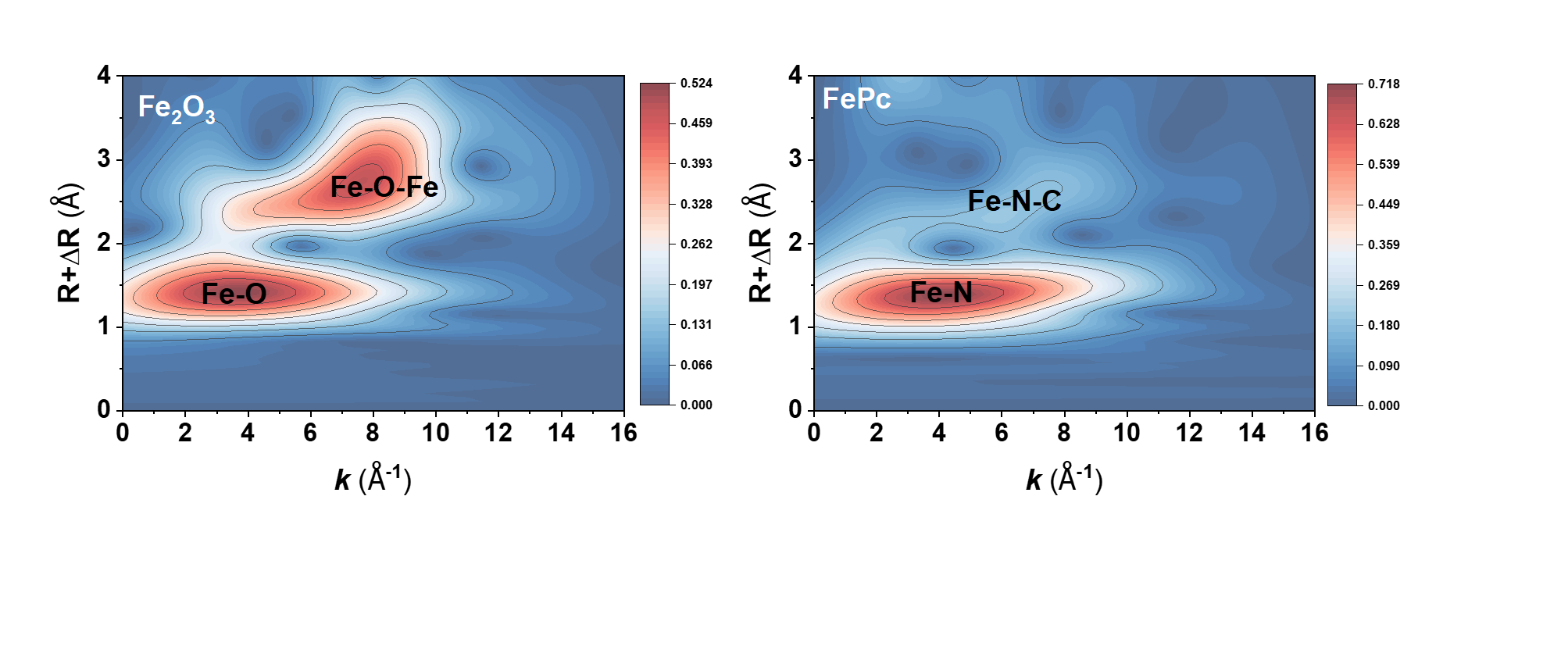


**Figure S7.** WT of Fe_2_O_3_ and FePc.


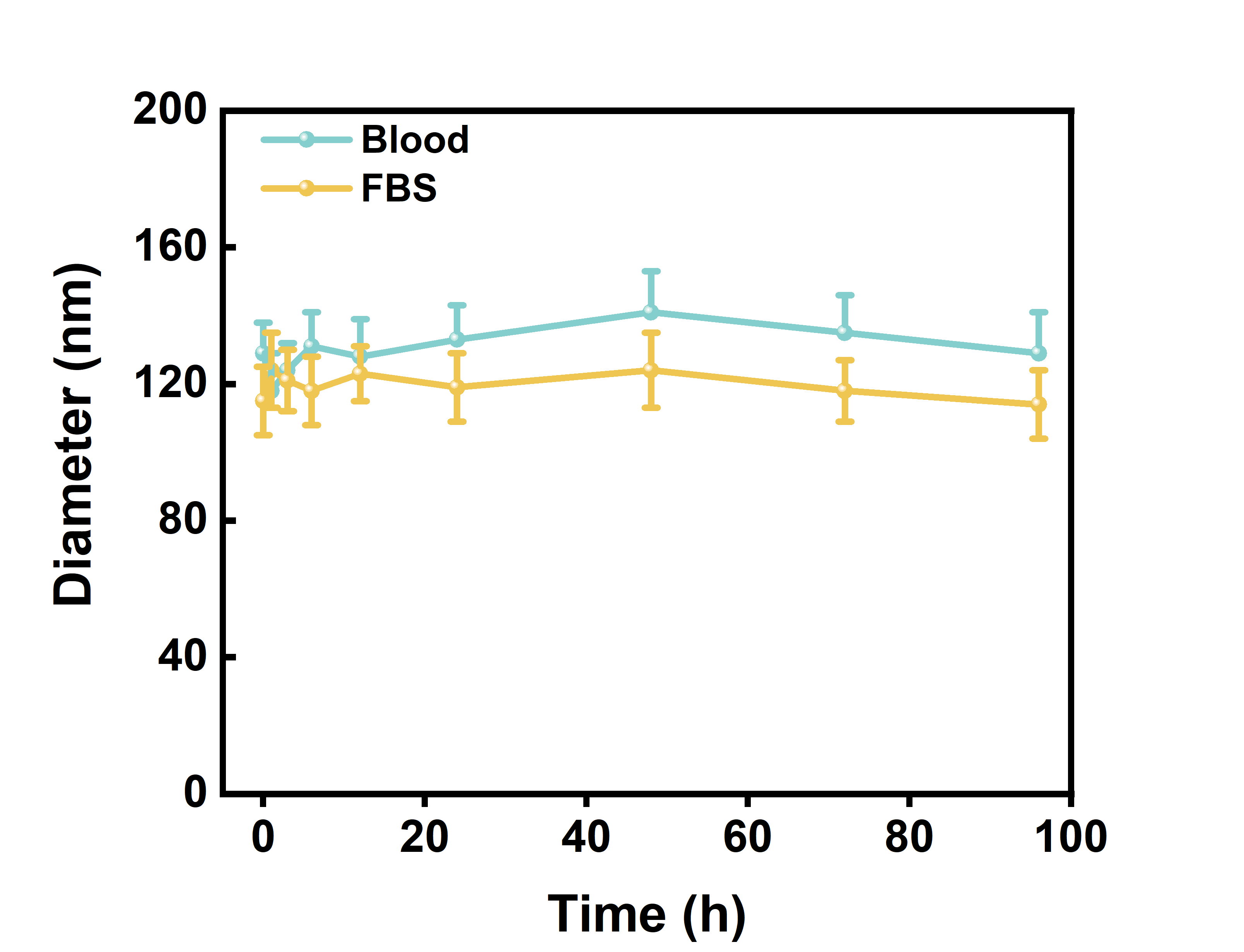


**Figure S8.** Hydrodynamic diameter of Fe-SiN_3_/SAN incubated in blood and fetal bovine serum (FBS) over 96 hours. Data are presented as mean ± standard deviation (n=3).

**
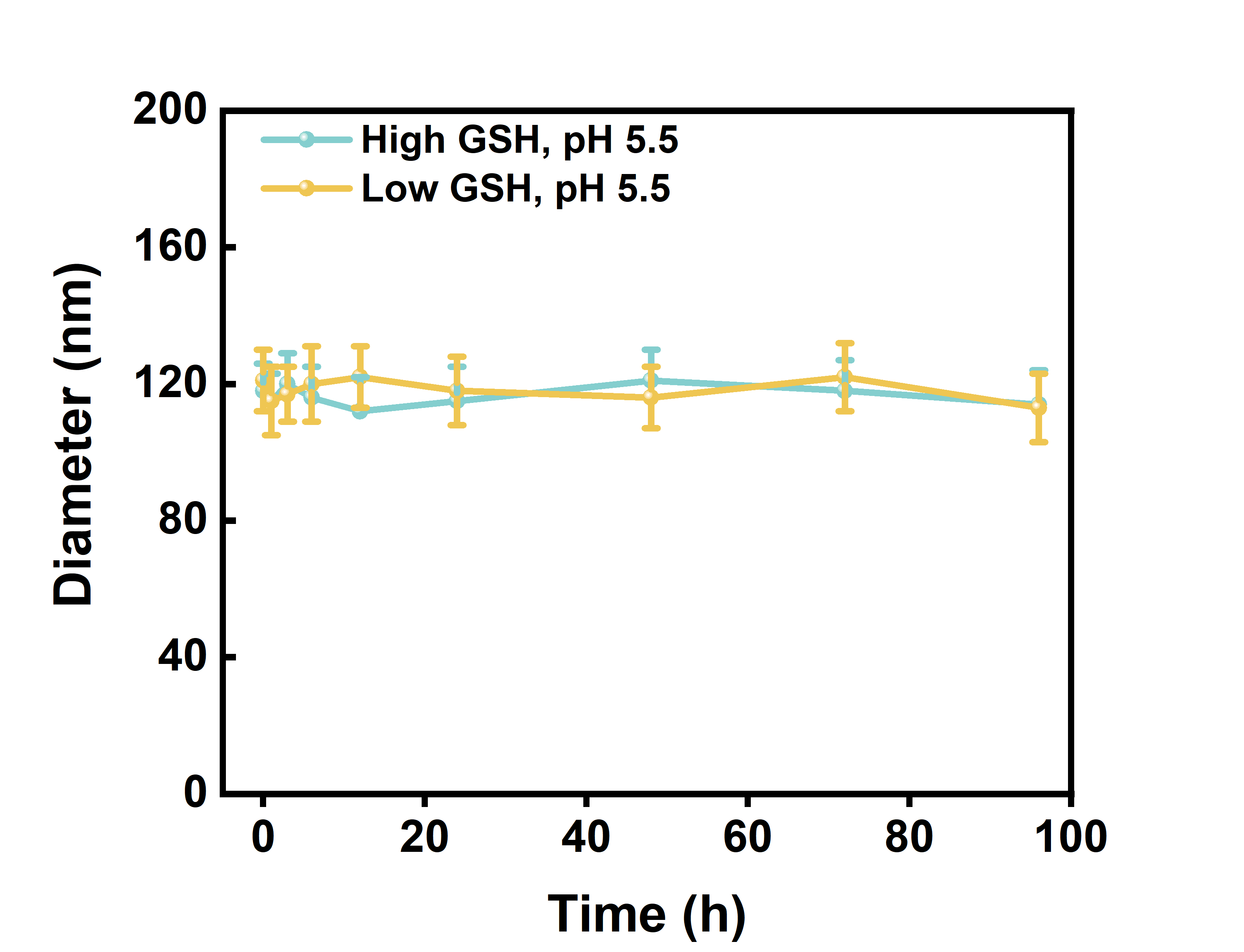
**

**Figure S9.** Hydrodynamic diameter of Fe-SiN_3_/SAN incubated in high GSH and low GSH over 96 hours. Data are presented as mean ± standard deviation (n=3).


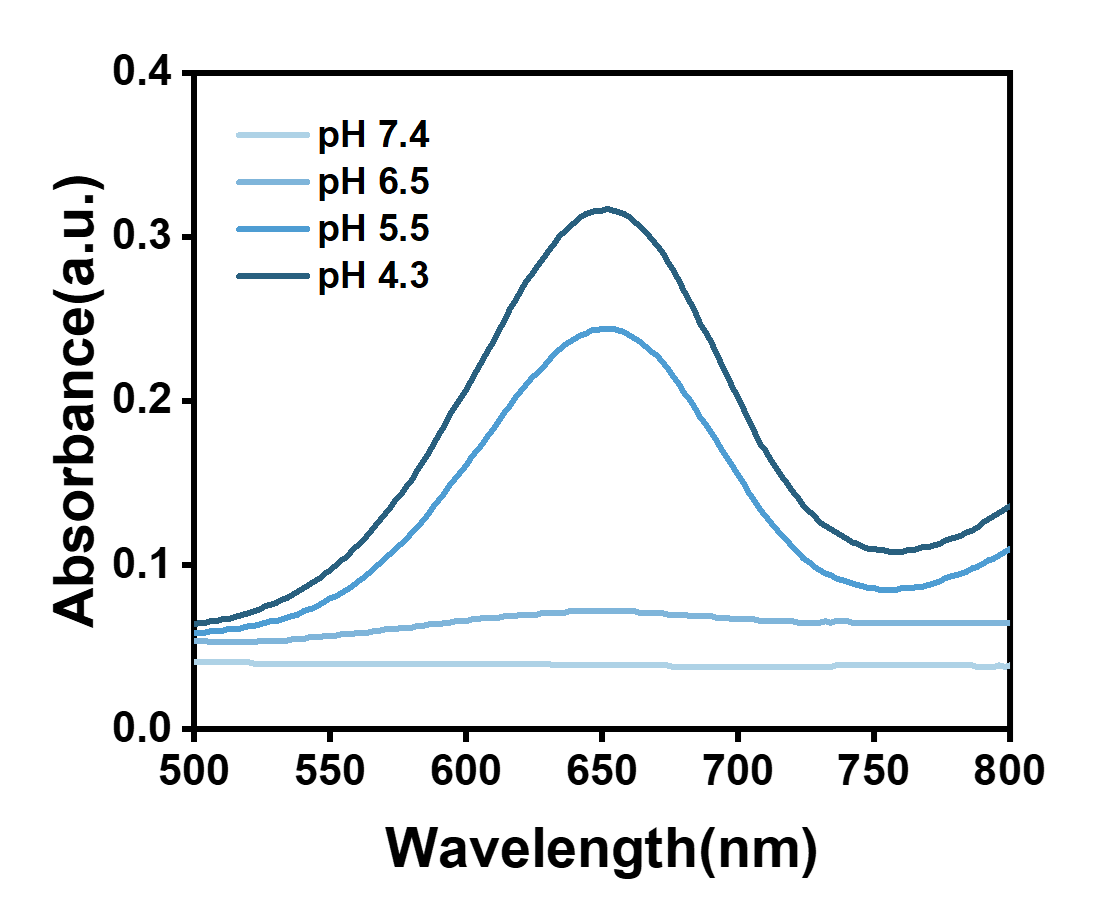


**Figure S10.** UV-vis spectra of TMB cultured with Fe-N4/SAN in the presence of H2O2 at various pH values.


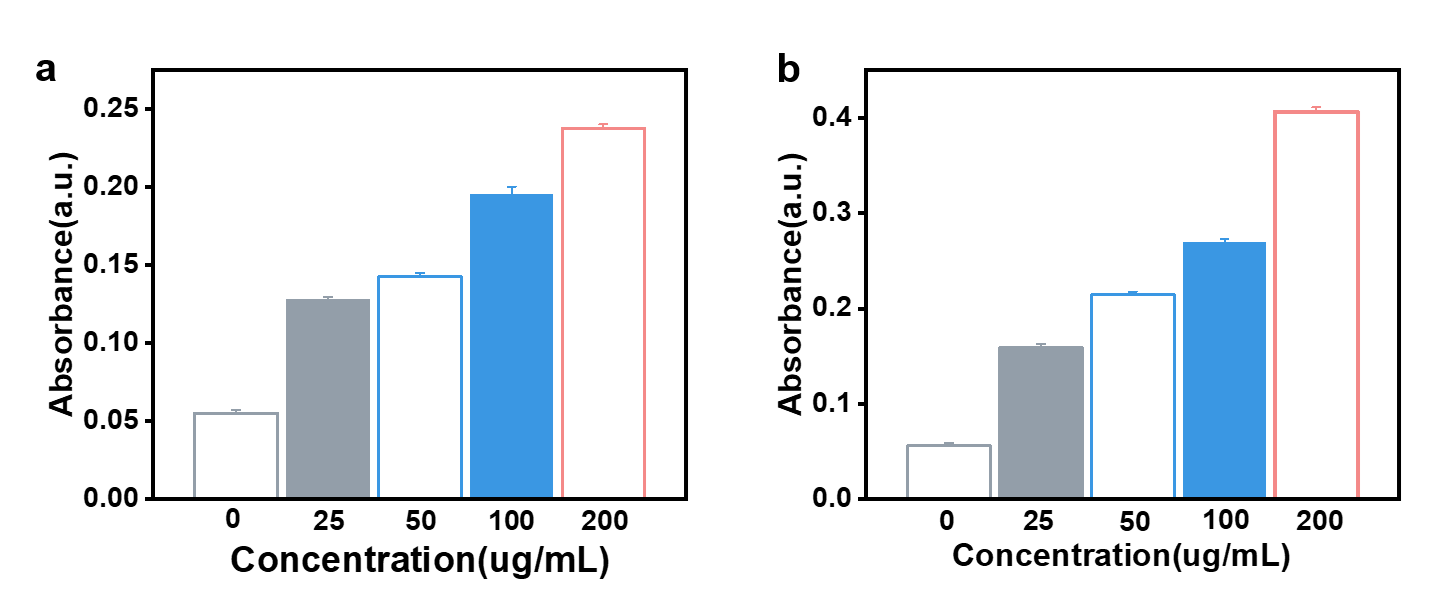


**Figure S11.** (a)TMB assay for measuring POD-like activity after incubating the different concentrations of Fe-N_4_/SAN and (b) Fe-SiN_3_/SAN.


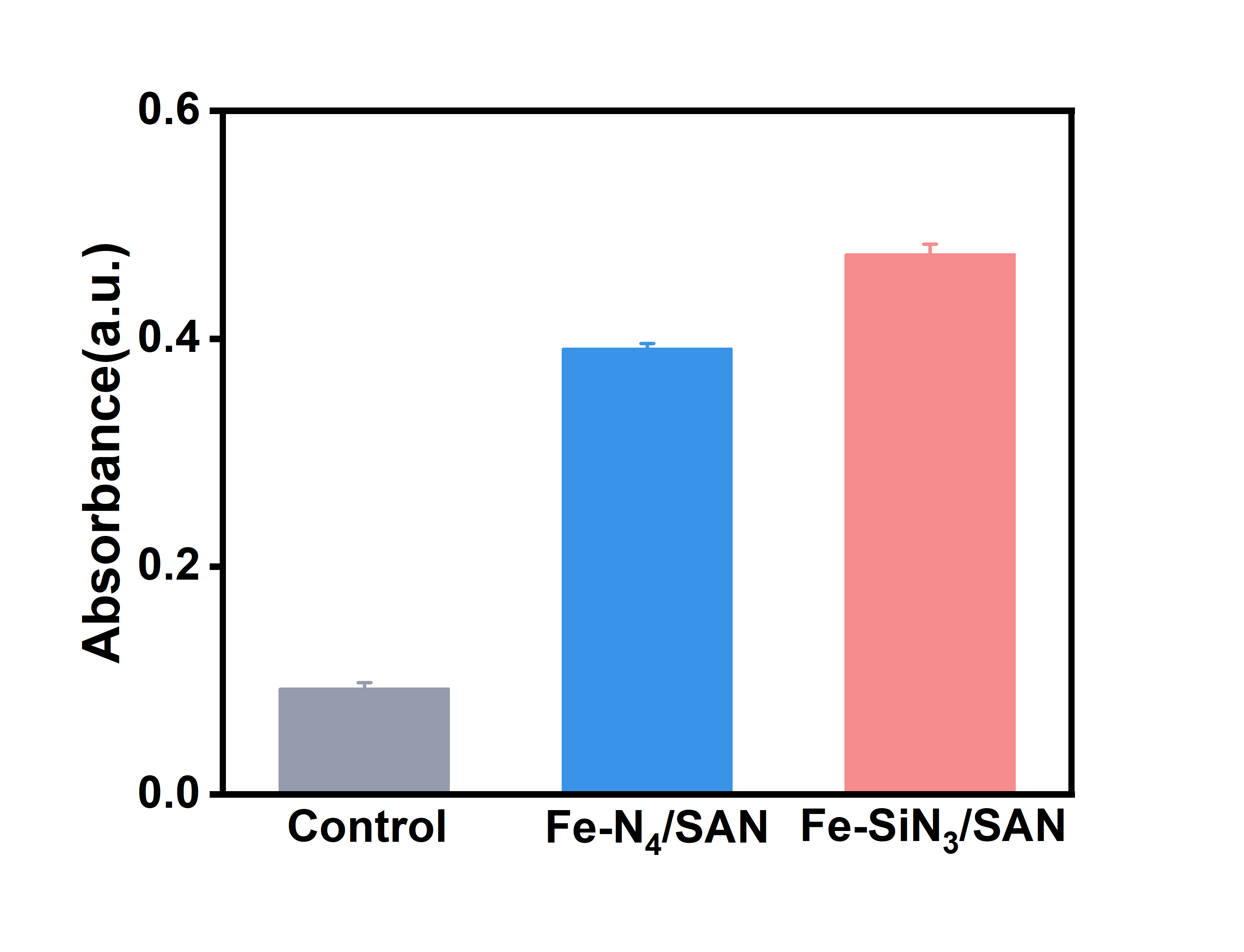


**Figure S12.** ABTS cultured with Fe-based SANs in the presence of H_2_O_2_ at acidic pH.

**
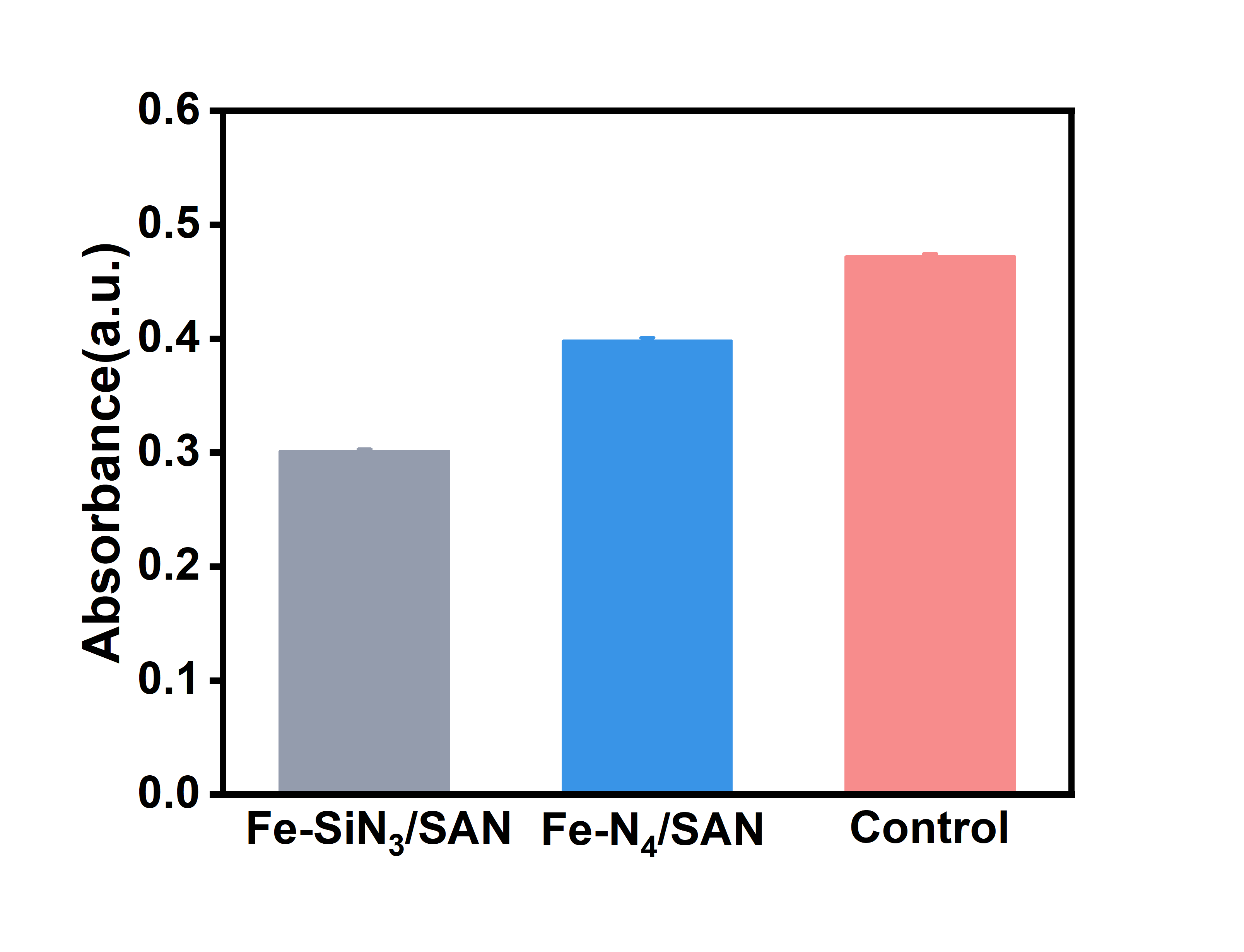
**

**Figure S13.** DTNB cultured with varying formulations in the presence of GSH.


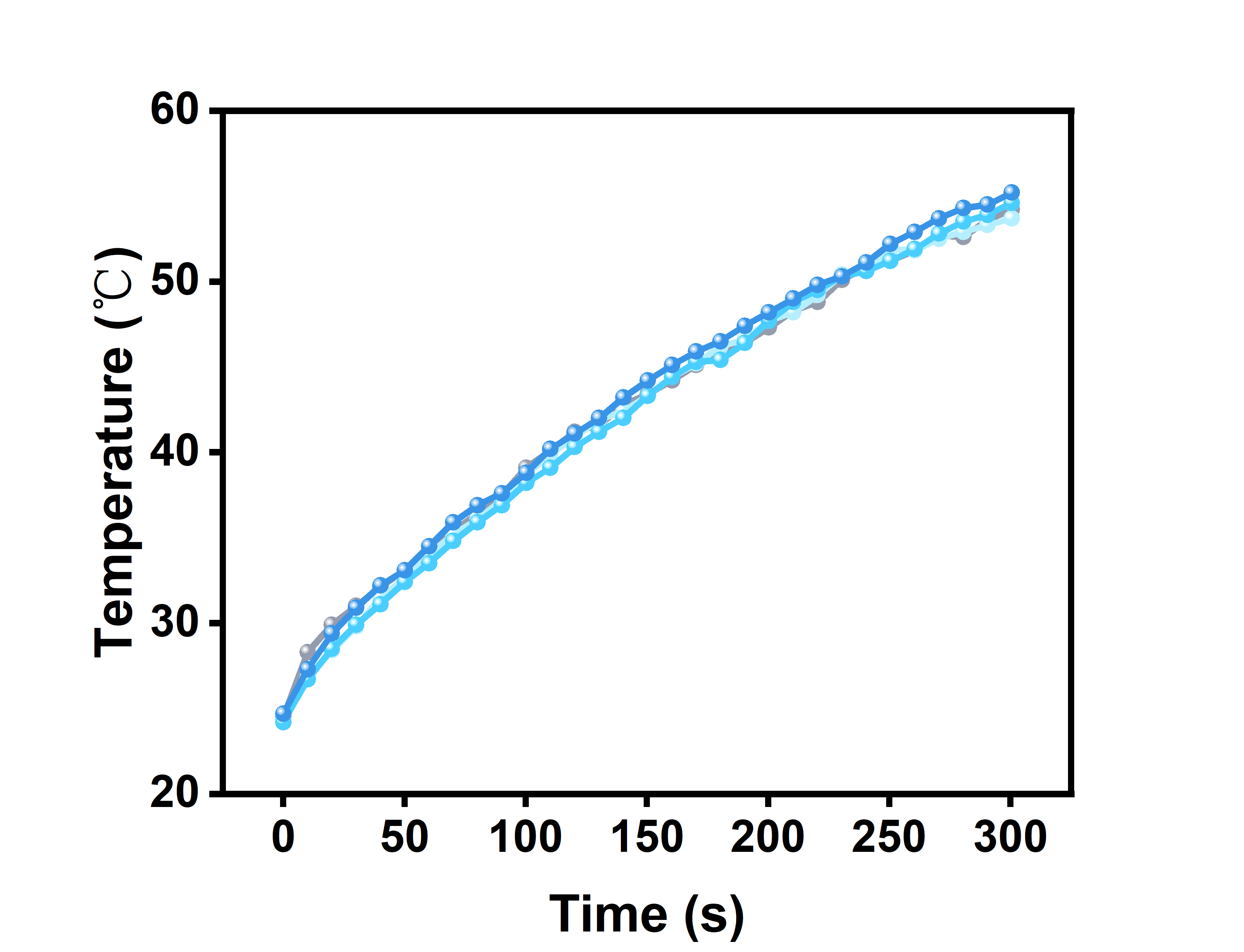


**Figure S14.** Fe-SiN_3_/SAN retain excellent thermal stability over four cycles of irradiation, as evidenced by consistent temperature profiles.

**
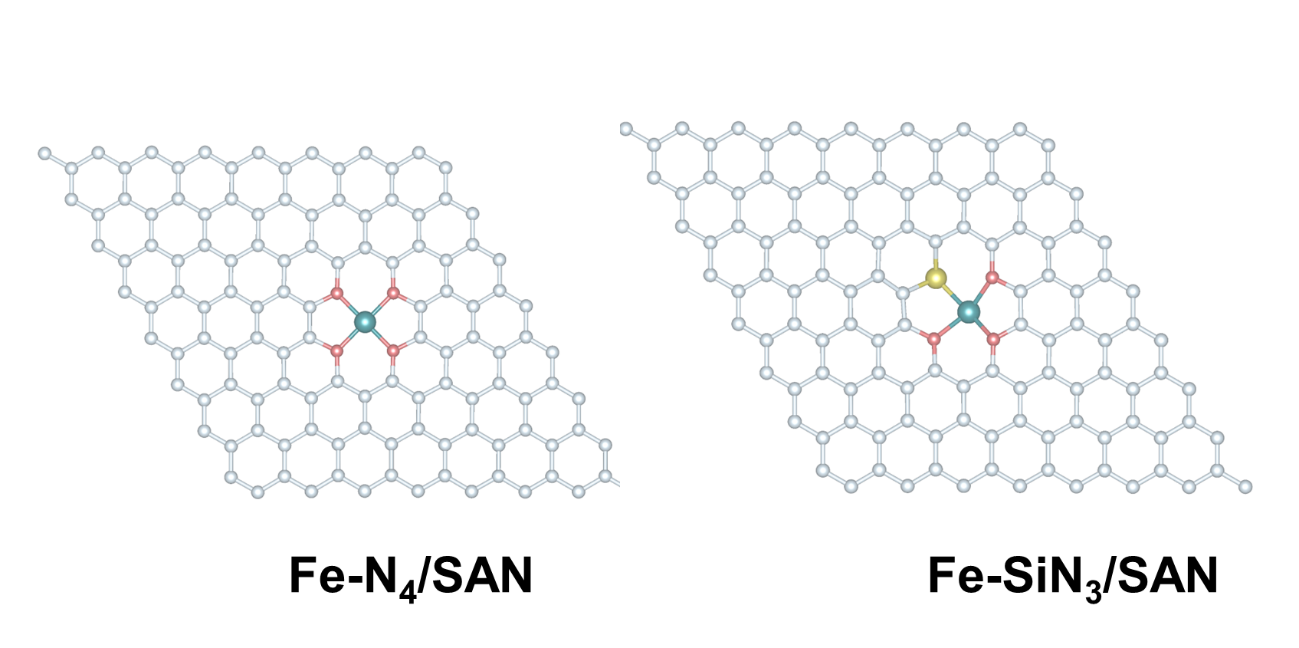
**

**Figure S15**. The optimistic structures of symmetric Fe-N_4_/SAN and asymmetric Fe-SiN_3_/SAN.

**
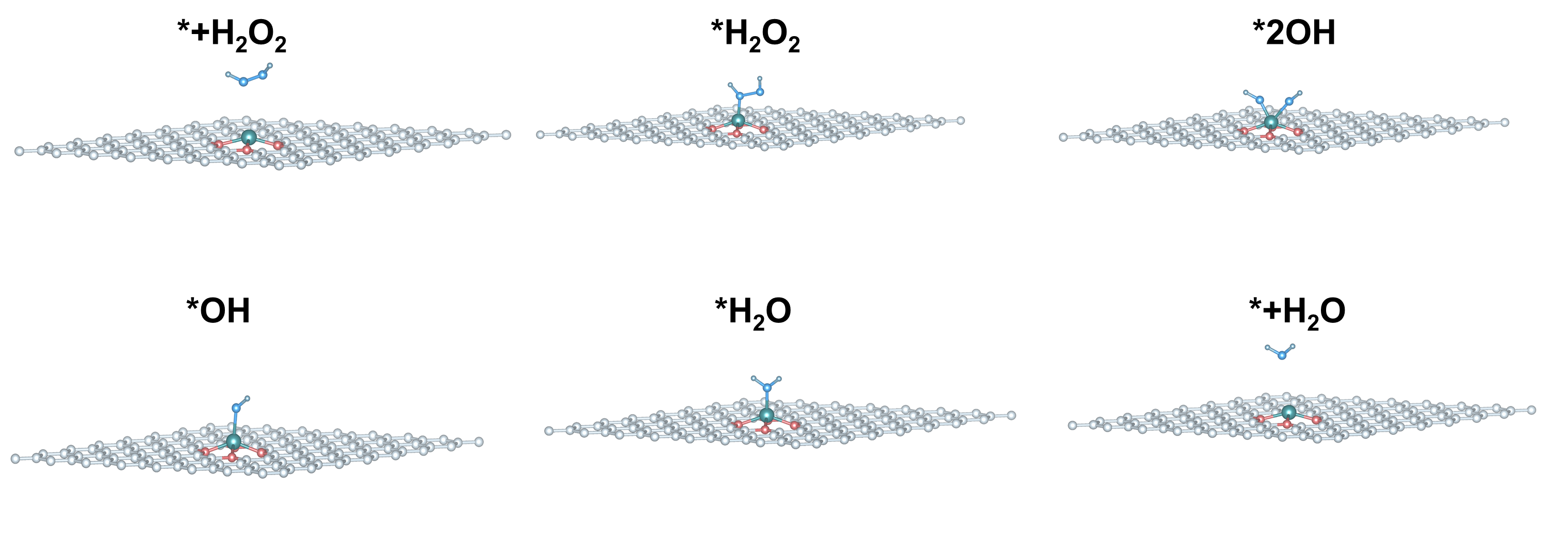
**

**Figure S16**. Gibbs free-energy diagrams for the decomposition of H_2_O_2_ into •OH on Fe-N_4_/SAN.

**
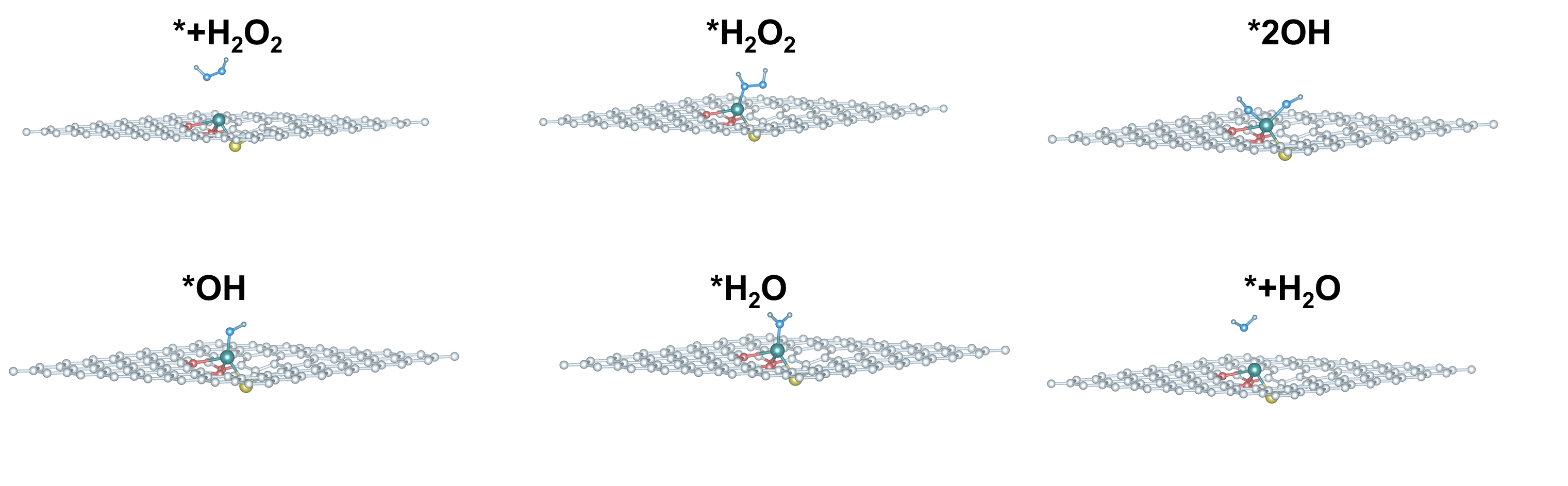
**

**Figure S17**. Gibbs free-energy diagrams for the decomposition of H_2_O_2_ into •OH on asymmetric Fe-SiN_3_/SAN.

**
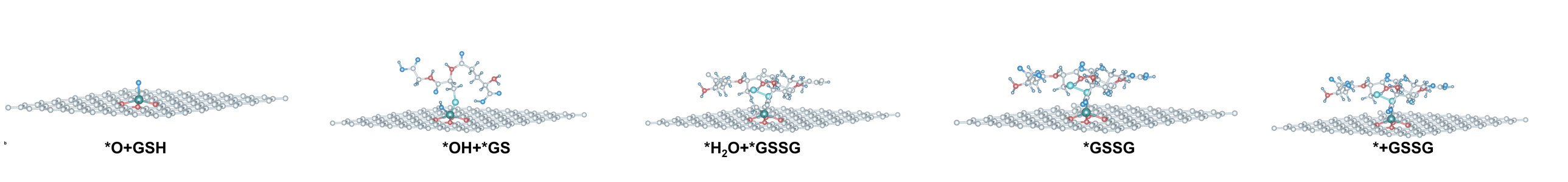
**

**Figure S18**. Gibbs free-energy diagrams for the decomposition of GSH into GSSG on Fe-N_4_/SAN.

**
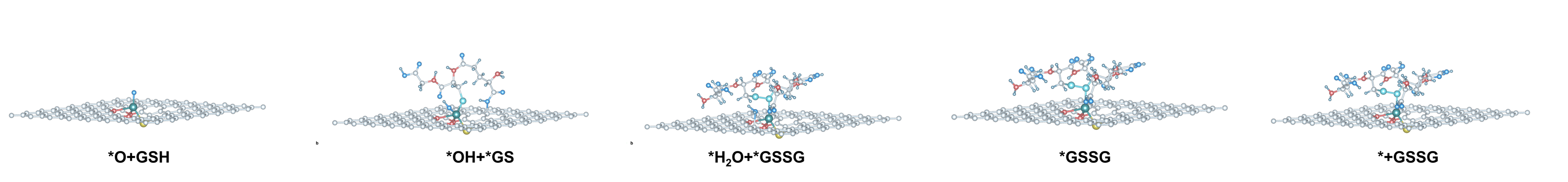
**

**Figure S19**. Gibbs free-energy diagrams for the decomposition of GSH into GSSG on asymmetric Fe-SiN_3_/SAN.


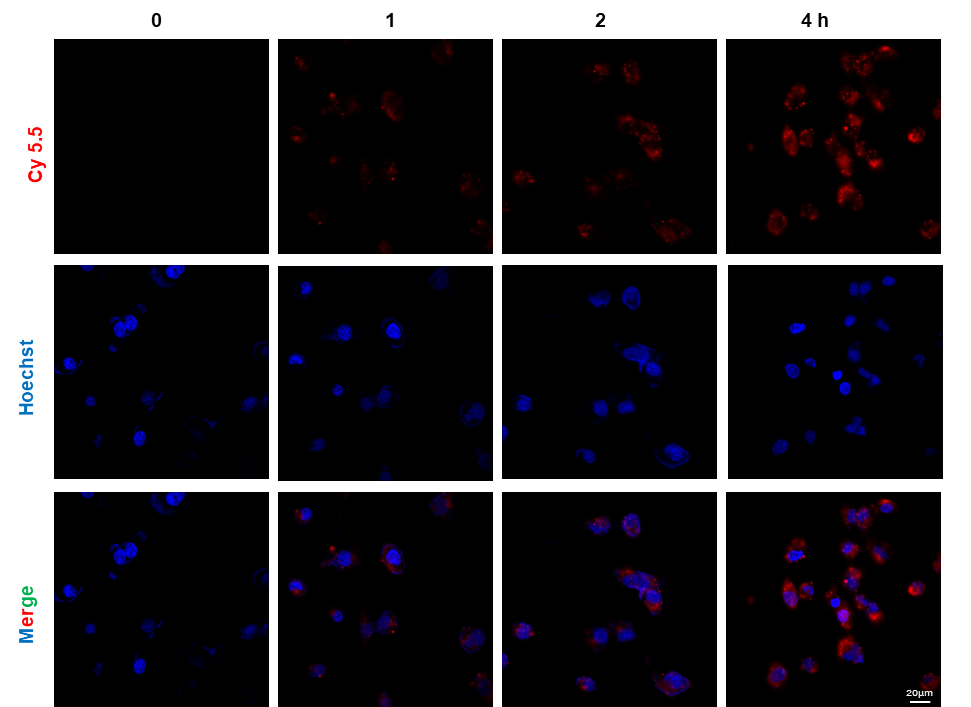
 **Figure S20**. The CLSM images of tumor cells incubated with Cy5.5-labeled Fe-SiN_3_/SAN.


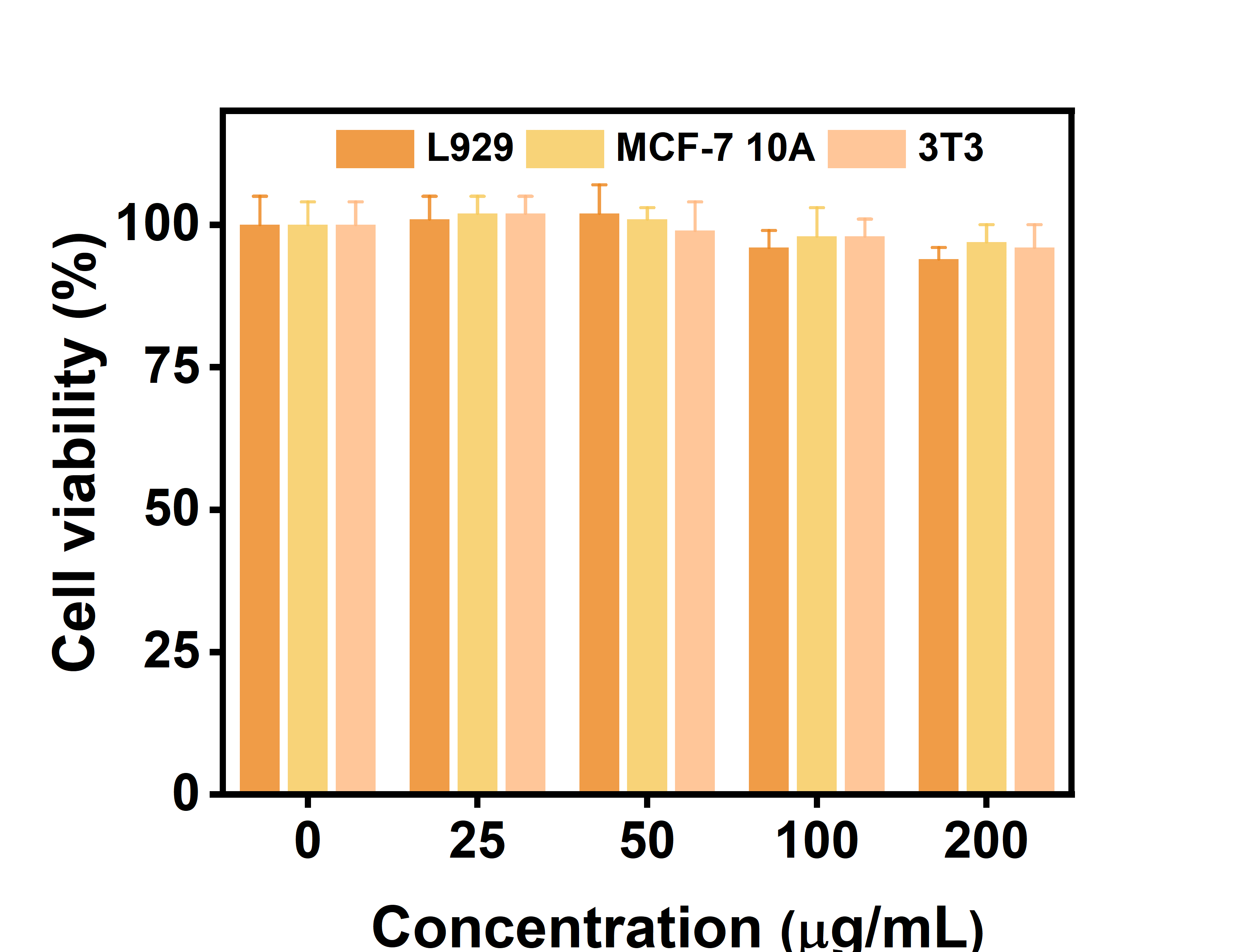


**Figure S21.** The anti-proliferation effect of Fe-SiN_3_/SAN on different normal cells.


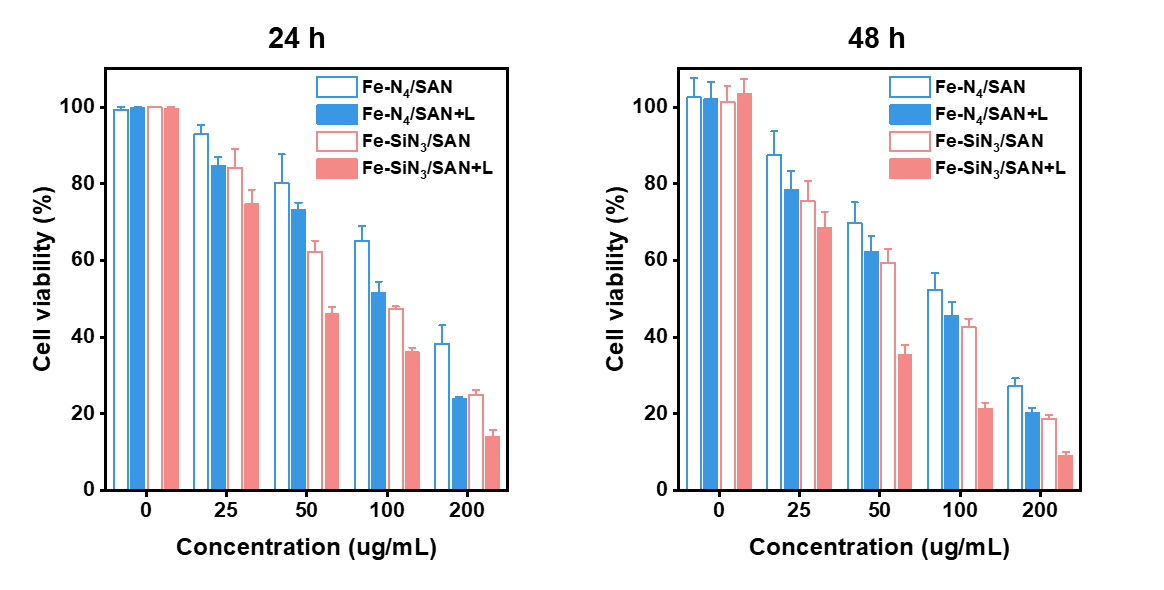


**Figure S22.** Tumor cell viability after 24 h and 48 h of incubation with varying concentrations of Fe-SiN_3_/SAN upon laser irradiation.


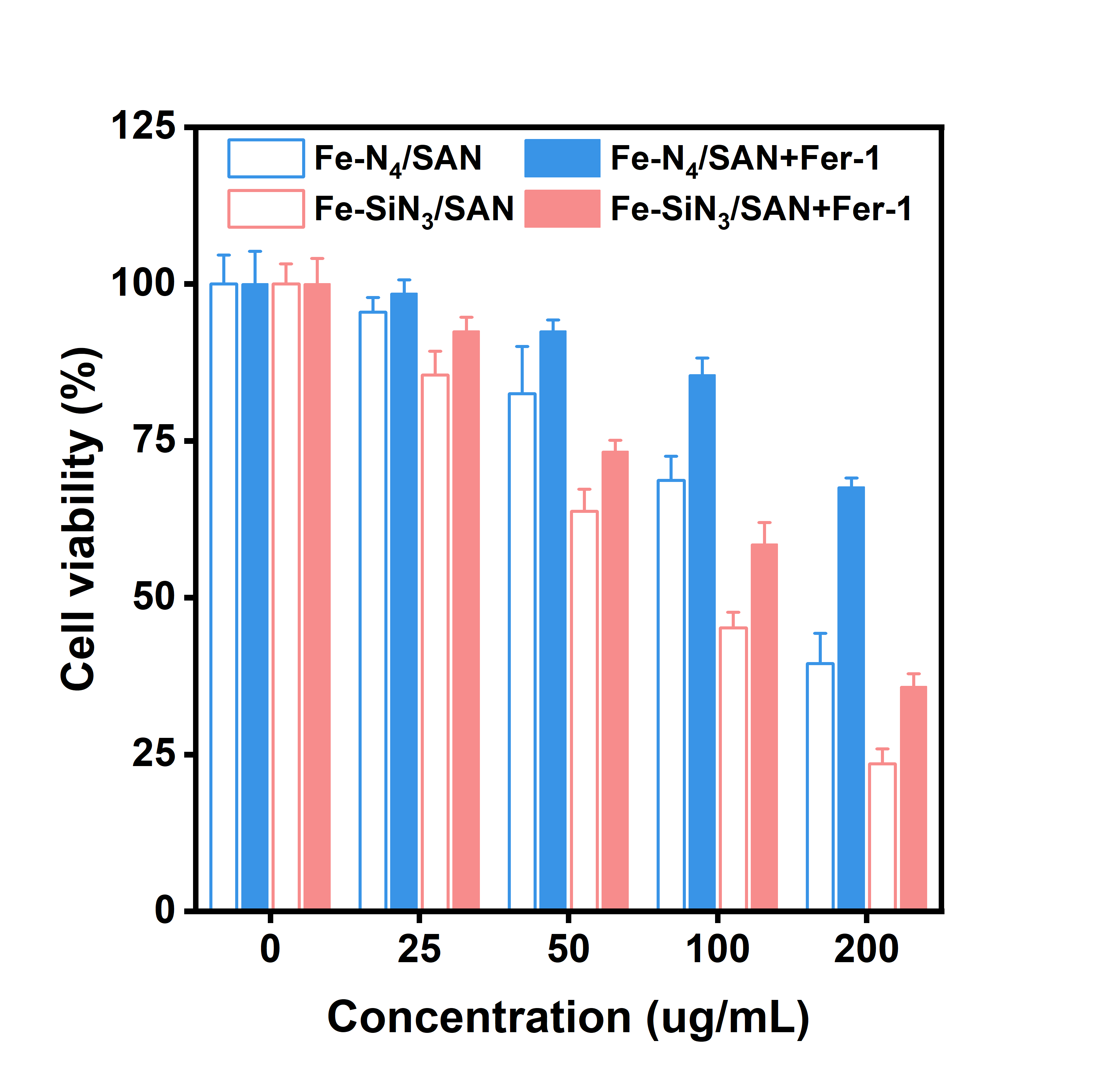


**Figure S23.** Tumor cell viability after 24 h of incubation with varying concentrations of Fe-N_4_/SAN or Fe-SiN_3_/SAN in the presence of Fer-1 (1 μM).


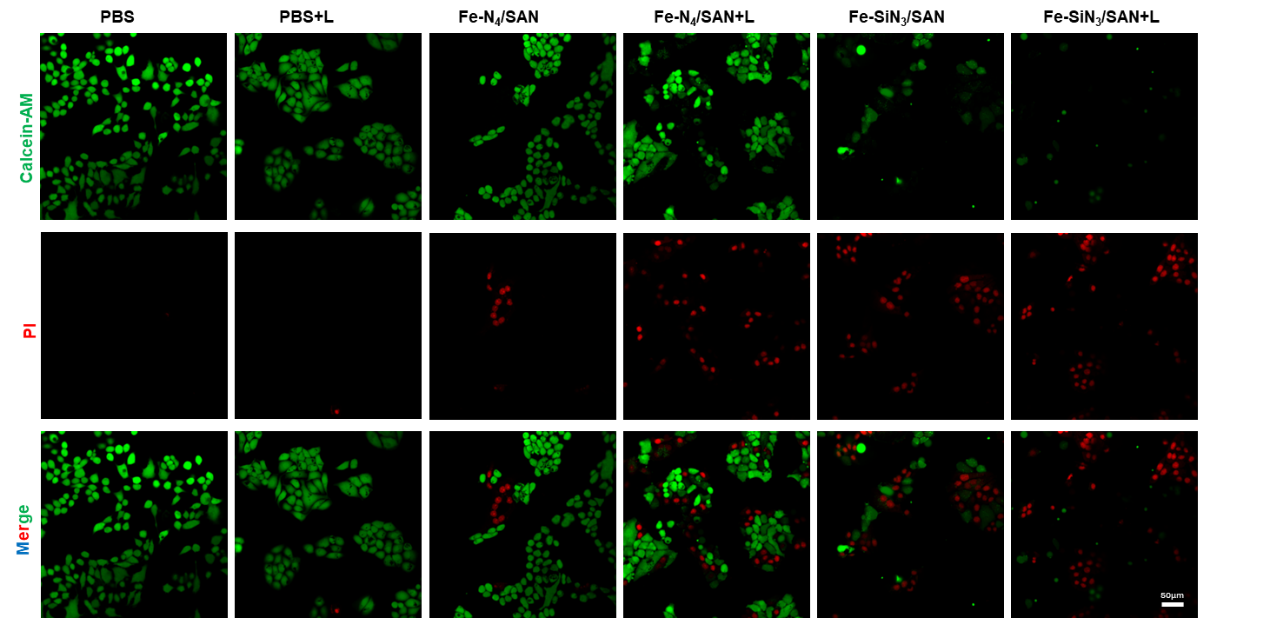
 **Figure S24**. Calcein-AM/PI co-stained Eca109 cells incubated with various formulations.


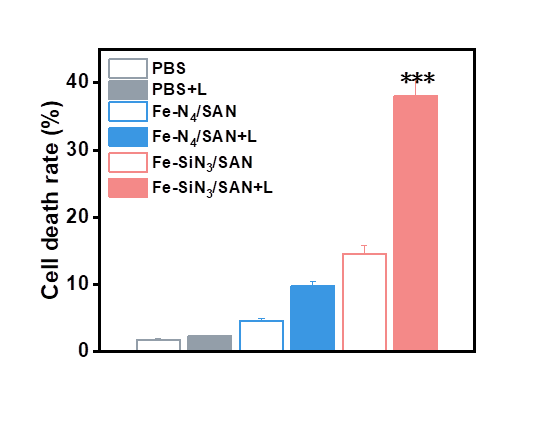


**Figure S25**. The proportion of cell death rates among different treatment groups


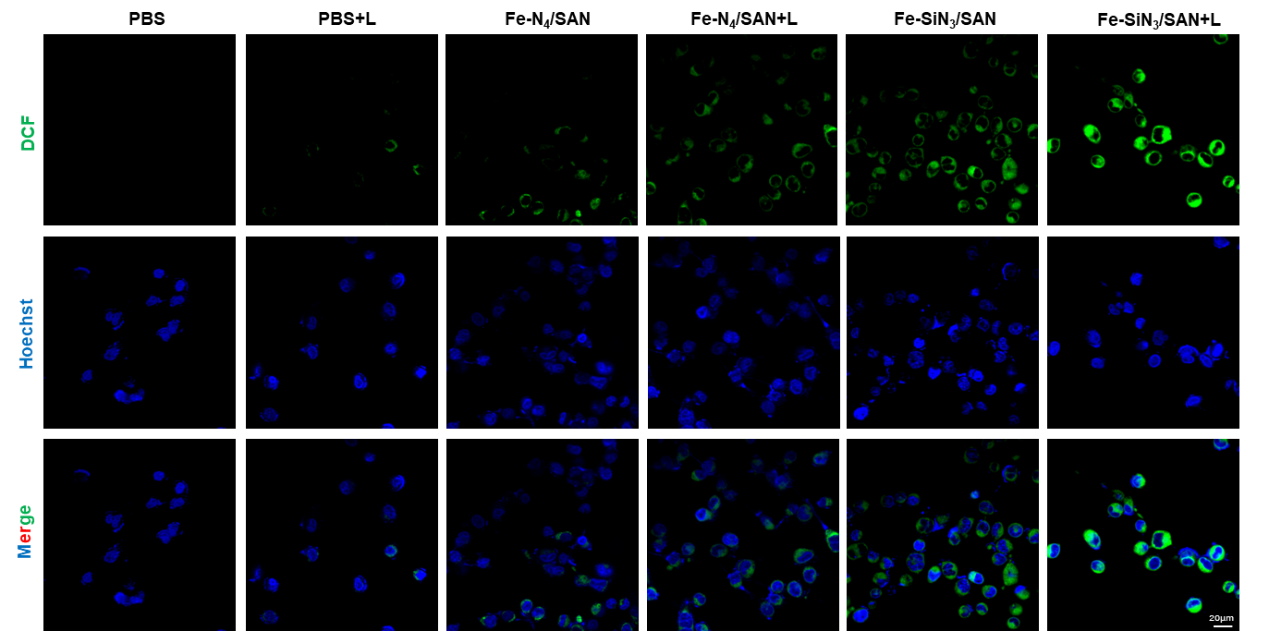


**Figure S26**. CLSM images of DCF in Eca109 cells treated with different formulations.


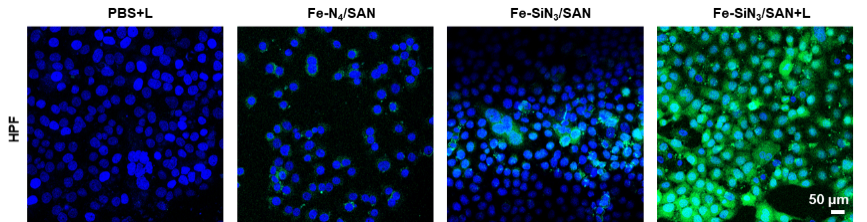


**Figure S27.** The fluorescence images •OH probe HPF-stained Eca109 cells following varying treatments.


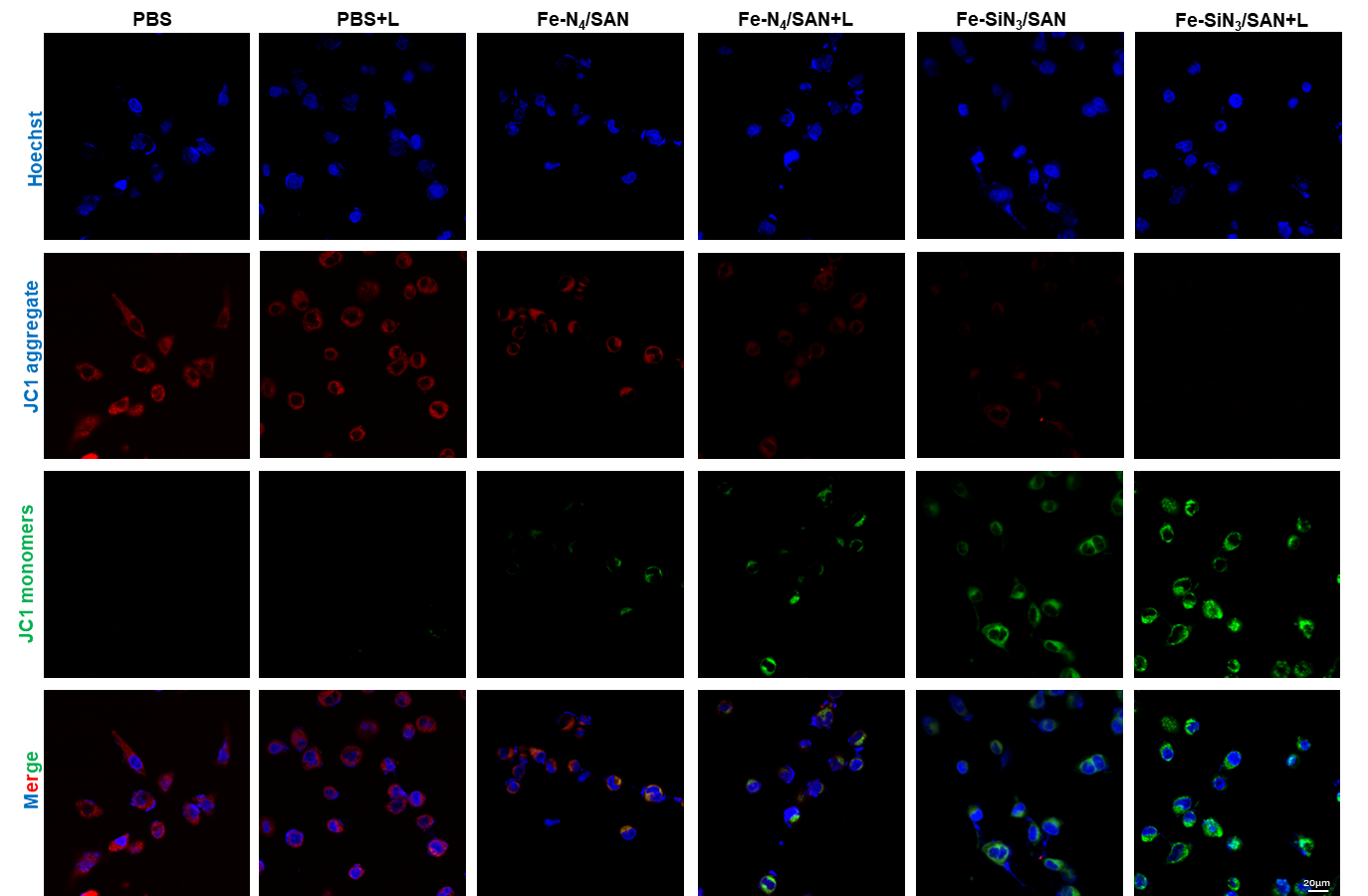


**Figure S28**. CLSM images of JC-1 in Eca109 cells treated with different formulations.


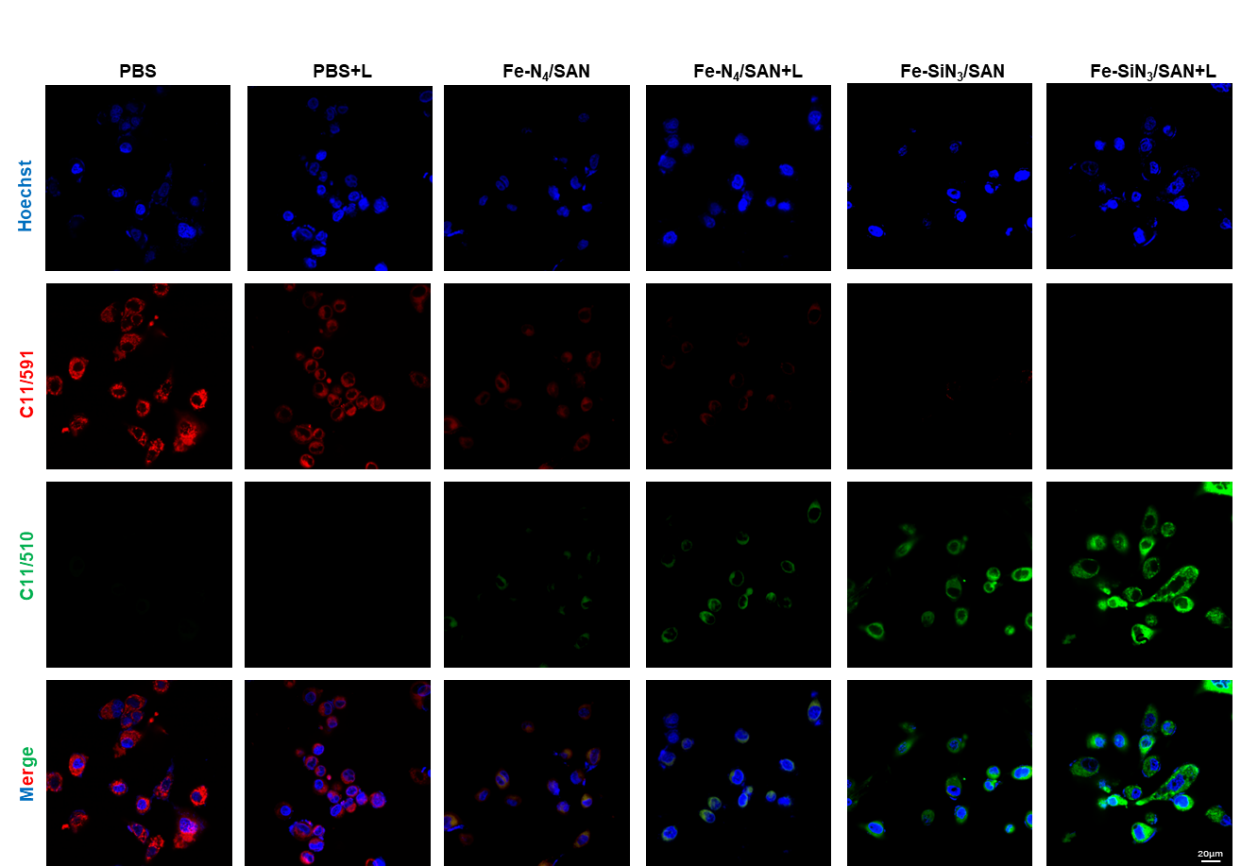


**Figure S29**. CLSM images of C11 BODIPY 581/591 in Eca109 cells treated with different formulations.


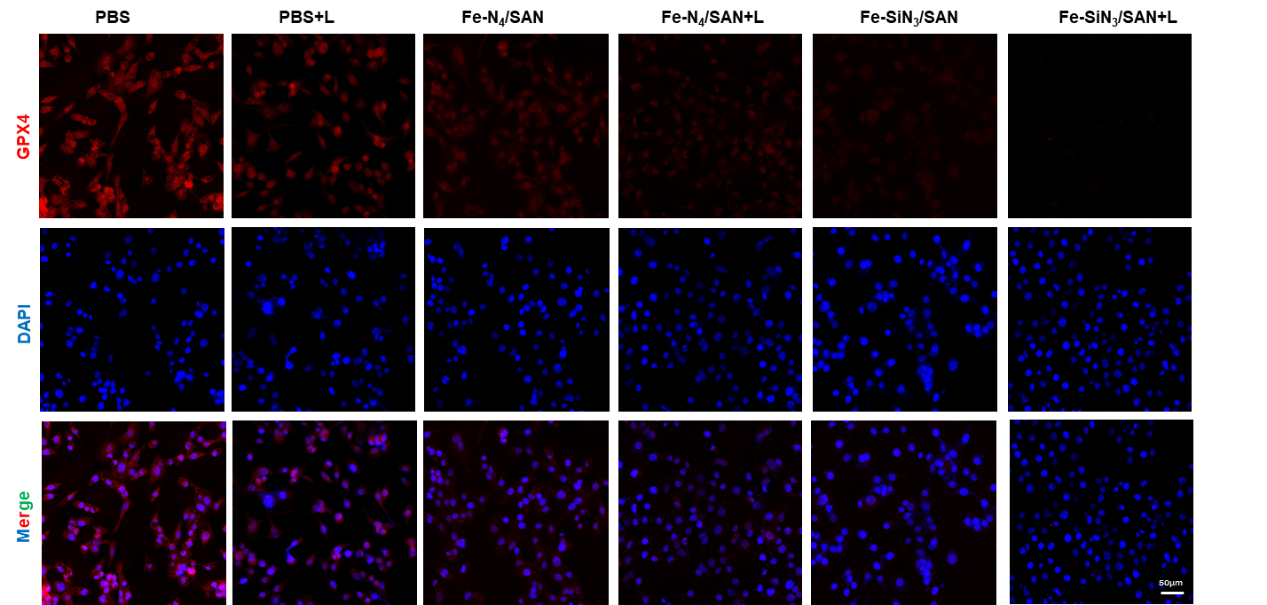


**Figure S30**. CLSM images of GPX4 expression in Eca109 cells treated with different formulations.


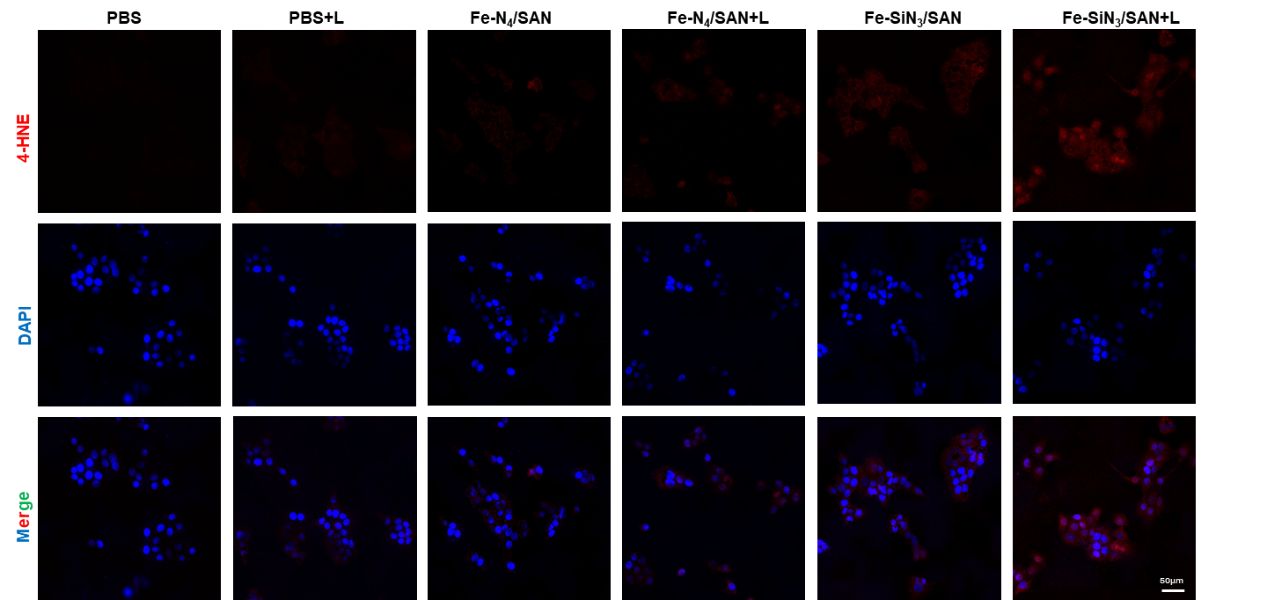


**Figure S31**. CLSM images of 4-HNE in Eca109 cells treated with different formulations.


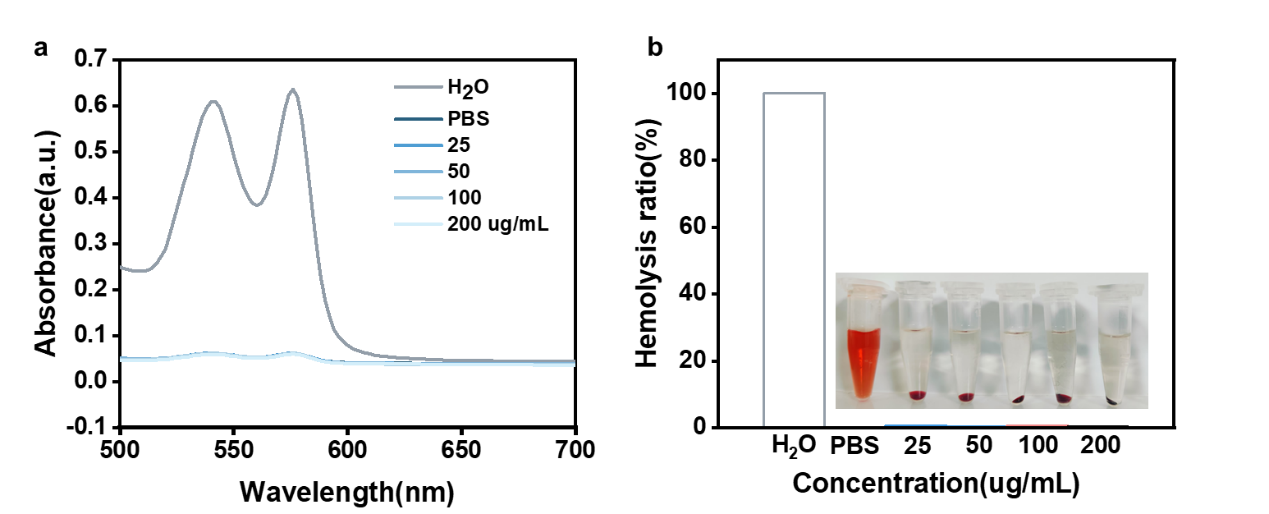


**Figure S32.** (a) Hemolysis analysis and (b) the corresponding quantification of red blood cells after treatment with various concentrations of Fe-SiN_3_/SAN.

**
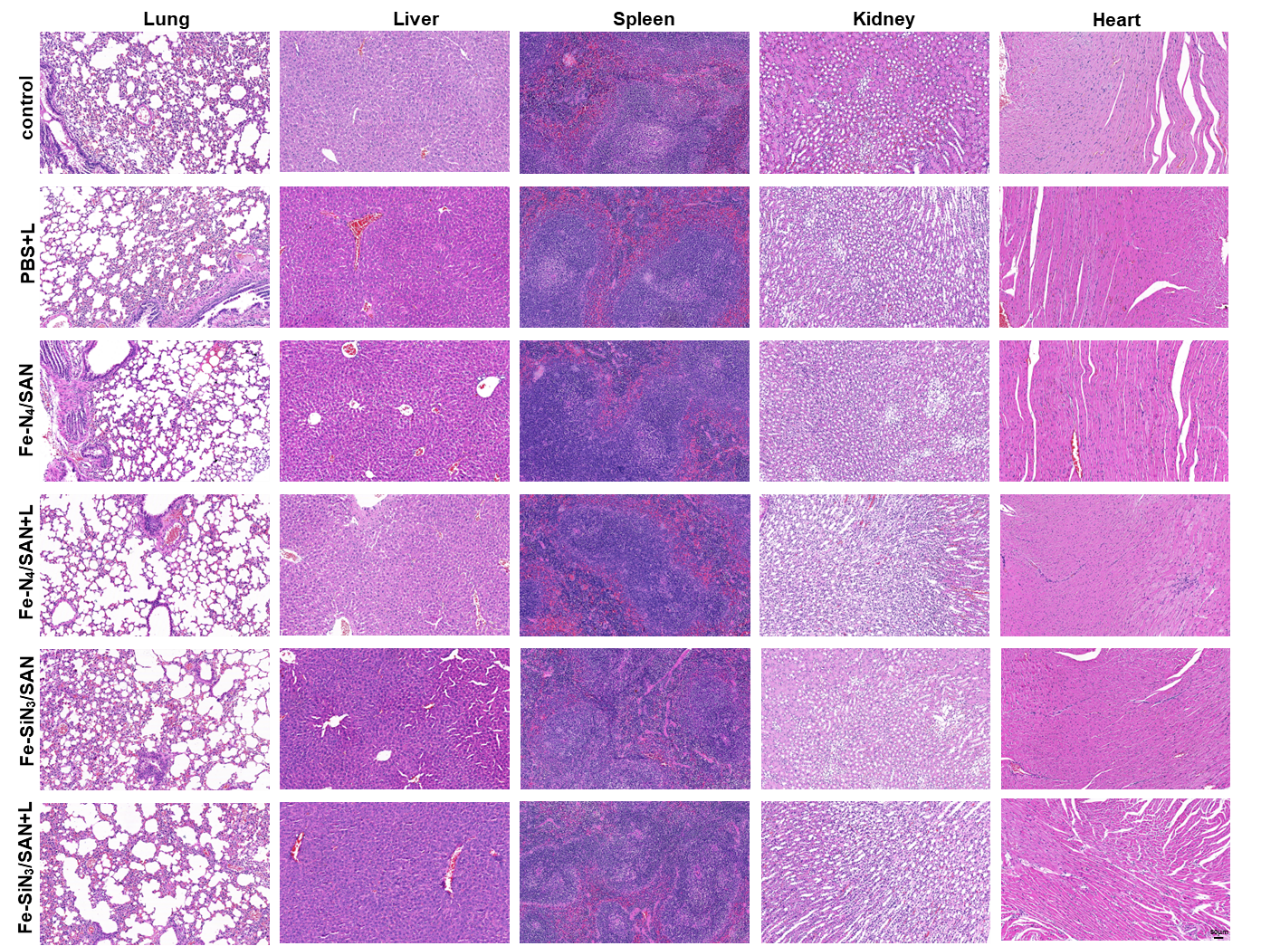
 Figure S33**. H&E staining of the major organs.


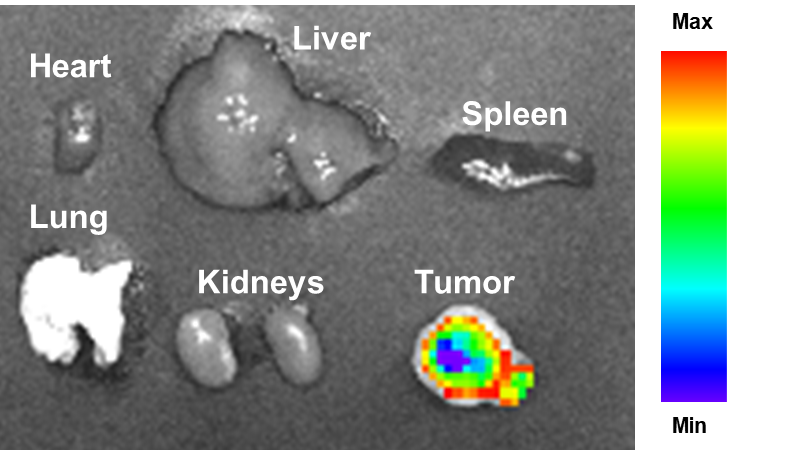


**Figure S34.** Fluorescence Imaging of major organs and tumor in mice.

**
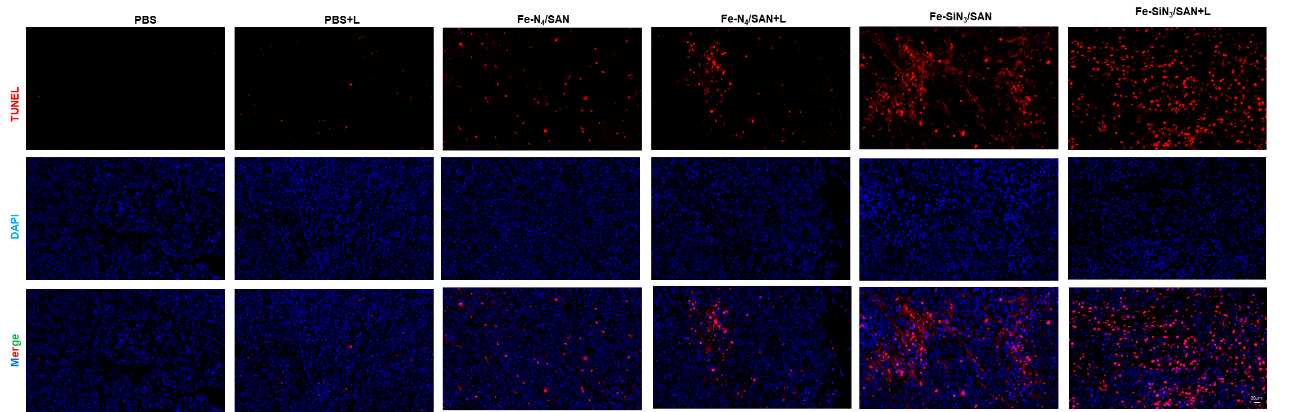
 Figure S35.** TUNEL staining of tumor tissue.


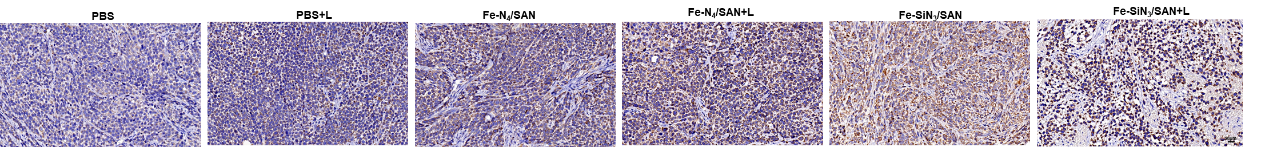


**Figure S36.** 4-HNE staining of tumor tissue.


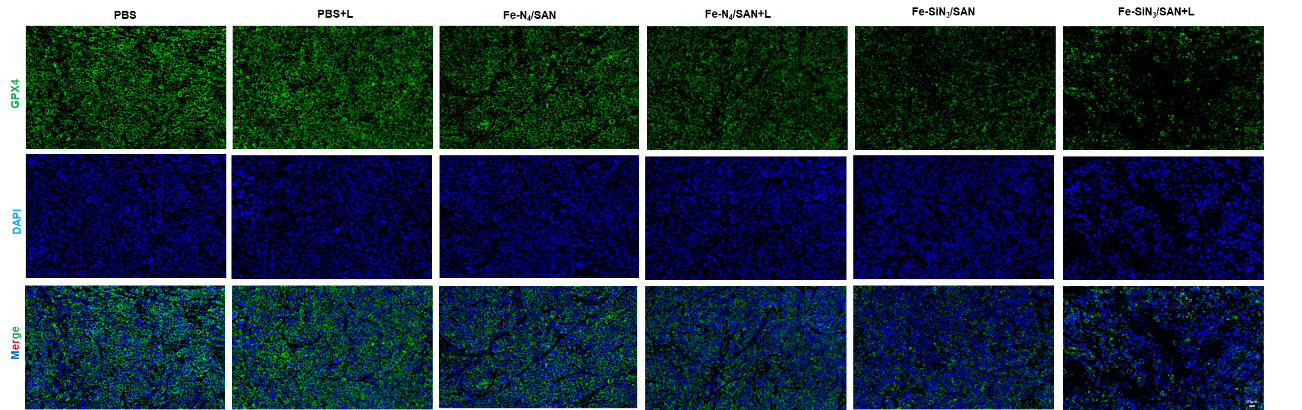


**Figure S37.** Immunofluorescence of GPX4 expression in tumor tissues following varying treatments.

**
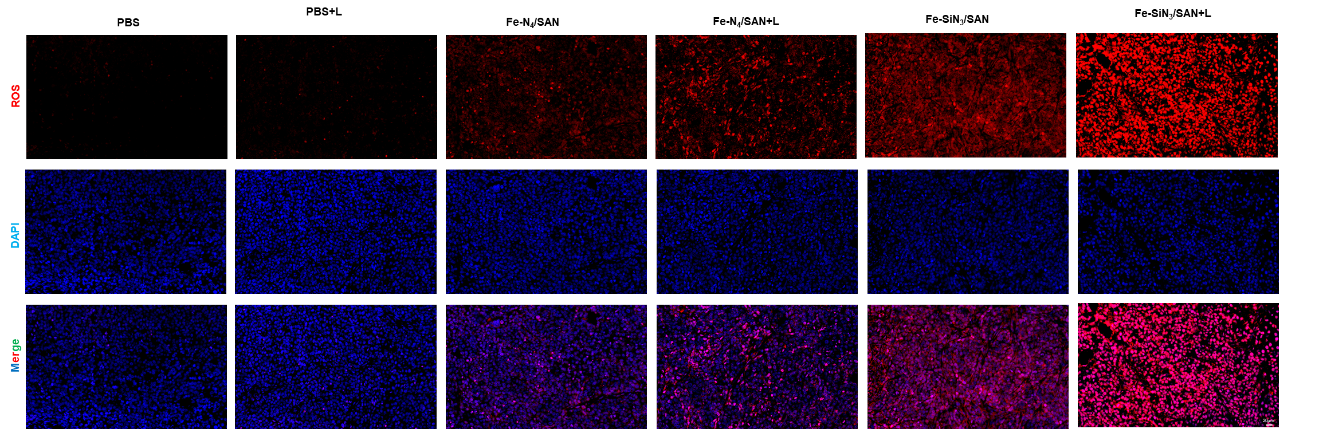
**

**Figure S38.** Immunofluorescence of ROS expression in tumor tissues following varying treatments.
